# Prospective cohort study of the relationship between neuro-cognition, social cognition and violence in forensic patients with schizophrenia and schizoaffective disorder

**DOI:** 10.1186/s12888-015-0548-0

**Published:** 2015-07-10

**Authors:** Ken O’Reilly, Gary Donohoe, Ciaran Coyle, Danny O’Sullivan, Arann Rowe, Mairead Losty, Tracey McDonagh, Lasairiona McGuinness, Yvette Ennis, Elizabeth Watts, Louise Brennan, Elizabeth Owens, Mary Davoren, Ronan Mullaney, Zareena Abidin, Harry G Kennedy

**Affiliations:** 1 Department of Psychiatry, Trinity College Dublin, Dublin, Ireland; 2 National Forensic Mental Health Service, Central Mental Hospital, Dundrum, Dublin 14, Ireland

**Keywords:** Schizophrenia, Violence, Mediation, MATRICS, MSCEIT, Neurocognition, Social cognition, Reasoning HCR-20, Function

## Abstract

**Background:**

There is a broad literature suggesting that cognitive difficulties are associated with violence across a variety of groups. Although neurocognitive and social cognitive deficits are core features of schizophrenia, evidence of a relationship between cognitive impairments and violence within this patient population has been mixed.

**Methods:**

We prospectively examined whether neurocognition and social cognition predicted inpatient violence amongst patients with schizophrenia and schizoaffective disorder (*n* = 89; 10 violent) over a 12 month period. Neurocognition and social cognition were assessed using the MATRICS Consensus Cognitive Battery (MCCB).

**Results:**

Using multivariate analysis neurocognition and social cognition variables could account for 34 % of the variance in violent incidents after controlling for age and gender. Scores on a social cognitive reasoning task (MSCEIT) were significantly lower for the violent compared to nonviolent group and produced the largest effect size. Mediation analysis showed that the relationship between neurocognition and violence was completely mediated by each of the following variables independently: social cognition (MSCEIT), symptoms (PANSS Total Score), social functioning (SOFAS) and violence proneness (HCR-20 Total Score). There was no evidence of a serial pathway between neurocognition and multiple mediators and violence, and only social cognition and violence proneness operated in parallel as significant mediators accounting for 46 % of the variance in violent incidents. There was also no evidence that neurocogniton mediated the relationship between any of these variables and violence.

**Conclusions:**

Of all the predictors examined, neurocognition was the only variable whose effects on violence consistently showed evidence of mediation. Neurocognition operates as a distal risk factor mediated through more proximal factors. Social cognition in contrast has a direct effect on violence independent of neurocognition, violence proneness and symptom severity. The neurocognitive impairment experienced by patients with schizophrenia spectrum disorders may create the foundation for the emergence of a range of risk factors for violence including deficits in social reasoning, symptoms, social functioning, and HCR-20 risk items, which in turn are causally related to violence.

**Electronic supplementary material:**

The online version of this article (doi:10.1186/s12888-015-0548-0) contains supplementary material, which is available to authorized users.

## Background

Most patients diagnosed with schizophrenia are never violent. However there is a small but significant association between schizophrenia and violence and with homicide in particular [1–3]. The relationship between violence and schizophrenia is thought to arise primarily from active symptoms such as delusions and co-morbid problems particularly substance misuse [1, 4]. But there is a link between schizophrenia and vulnerability to substance misuse and an increased risk of violence remains even when substance misuse is taken into account [4, 5]. Also violent acts carried out by people with schizophrenia are complex and cannot always be explained by psychotic symptoms alone. Some people with schizophrenia can become violent at a young age prior to the onset of psychosis, whereas others become chronically violent after the first psychotic episode even when receiving medication, and there are those who commit only a single act of violence during their lifetime [1, 3, 6]. Furthermore the violent acts carried out by people with schizophrenia appear to be driven by some of the same risk factors as violence in general [6–9]. Violence risk prediction schemes such as the Historical-Clinical-Risk-20 (HCR-20) [10, 11] take advantage of this and assess violence proneness by including a large number of equally weighted items [12] that are not specific to schizophrenia or mental disorder but are associated with suboptimal functioning. For example, substance misuse, homelessness, employment problems, relationship problems, lack of social support, history of victimisation and criminal history, are all risk factors for violence [13–15]. Many of these difficulties are likely to be underpinned by the cognitive decline experienced by patients with schizophrenia [16–20]. Neurocognitive impairments may therefore represent a common or distal risk factor whose influence on violence is mediated by a range of more proximal risk factors.

### Impaired neurocognition and social cognition in schizophrenia

Although not a core diagnostic feature in DSM-5 [21] or ICD-10 [22], cognitive impairment has always been associated with schizophrenia [17, 23, 24]. Contemporary research has quantified this association using a range of neuropsychological tasks. On these measures patients with schizophrenia perform worse than healthy controls by as much as 2 standard deviations [17]. These impairments are thought to occur prior to the onset of psychosis. [17]. Crucially the problems also occur in medication naïve patients [17]. Standardised batteries have been developed to assess the cognitive problems experienced by patients with schizophrenia, of which the Measurement and Treatment Research to Improve Cognition in Schizophrenia (MATRICS) Consensus Cognitive Battery (MCCB) is one example [25]. The cognitive tasks on which patients perform poorly include not only neuropsychological or neurocognitive tests of memory, attention, and executive functioning, but also tests of social cognition such as perception of affect, emotional awareness, theory of mind, context sensitive processing, and emotional reasoning. [26]. Like neurocognitive deficits, many of these social cognitive problems are thought to be stable across phases of illness and linked to suboptimal functioning [17, 27]. For example, three tests - emotional reasoning (using the Mayer-Salovey-Caruso Emotional Intelligence Test MSCEIT), theory of mind and social relationship perception all predicted real world functioning at twelve months for patients experiencing first episode psychosis [28]. Social cognitive problems appear to account for additional variance of real world social functioning even when controlling for neurocognition [29]. Recent evidence also suggests that deficits in social cognition may mediate the relationship between neurocognitive impairments and positive symptoms, which have traditionally been seen as two separate domains [27, 30]. Because of the importance of the construct of social cognition for real world functioning and because of its strong psychometric properties, the managing emotion branch of the MSCEIT was included as a separate domain within the MCCB [25]. Finally both neurocognitive and social cognitive problems represent a major source of disability for patients with schizophrenia, accounting for more of the variance in functional outcome than symptoms [17, 29]. Patients with severe cognitive impairments have difficulties functioning day to day, finding meaningful employment and living independently [17].

### Impaired neurocognition and violence in schizophrenia

An association between neurocognition and violence has been documented in meta-analyses and reviews concerning brain injury, delinquency, and intellectual disability even when controlling for genetic and socioeconomic factors [31–33]. In contrast findings from the schizophrenia and violence literature are contradictory and harder to interpret. One recent meta-analysis failed to support a relationship between psychosis, neurocognition and violence [15]. The analysis examined a variety of cognitive factors including lower total scores on the full scale Wechsler Adult Intelligence Scale (WAIS), lower scores on the verbal subscale of the WAIS, lower scores on the performance subscale of the WAIS, lower total scores on the National Adult Reading Test (NART), and poorer executive functioning (higher perseverative errors on the Wisconsin Card Sorting Test). However, Witt et al. [15] advised caution in ruling out a relationship between cognition and violence because of the large amount of case studies suggesting a link and also because other systematic reviews have identified that theory of mind, insight and attitudinal cognition may be risk factors for violence [14]. In addition, two other recent literature reviews exploring the relationship between cognition and violence produced equivocal findings [3, 34]. None of the studies reviewed assessed the range of neurocognitive deficits associated with schizophrenia as outlined in the MATRICS consensus battery.

### Impaired social cognition and violence in schizophrenia

In comparison with neurocognitive deficits, problems with social cognition are likely to be particularly relevant to violence risk [14]. But because social cognition is also a multidimensional construct a variety of measures have been developed to measure these processes [35]. Social cognitive processes are also thought to occur in an informational processing stream with perception of affect and emotional awareness occurring before more abstract processes such as emotional reasoning [26]. Many of the constructs which fall under the social cognitive umbrella have their own historical roots and have grown out of a variety of literatures. For example it is possible to make distinctions between the constructs of theory of mind, mentalisation and empathy [36–38]. Theory of mind, the ability to attribute mental states to oneself and to others and the realisation that others have mental states different from one’s own is primarily associated with the field of autism research. Mentalisation, the ability to understand mental states when one’s attachment system is activated has its roots within the psychodynamic, borderline personality disorder and attachment literature. Empathy undoubtedly involves theory of mind but also includes the ability to experience a compassionate emotional response in relation to another’s suffering, and is primarily associated with developmental and social psychology. Theory of mind, mentalisation and empathy have all been related to violence in schizophrenia [39]. However because research on social cognition and schizophrenia is in its infancy there have been difficulties developing psychometrically sound and agreed upon instruments for measuring different components of social cognition [25]. In particular it has been challenging to measure empathy in schizophrenia in part due to the limitations of self-report questionnaires [40]. It was for this reason the managing emotions branch of the MSCEIT was the only social cognitive measure to be selected for use within the consensus battery of cognitive deficits in schizophrenia [25].

### Instrumental and reactive violence in schizophrenia

Few of the studies exploring the relationship between cognition and violence in schizophrenia have included measures of social reasoning or made a distinction between instrumental and reactive violence. Instrumental violence is predatory, goal directed and complex requiring forethought and sequential planning, whereas reactive violence is impulsive, defensive and executively simple [41–44]. Cognitive scientists have argued that reason, judgement, and decision making are not adequately measured by intelligence tests and are distinct domains of ability [45]. Impaired ability to foresee potential outcomes and to weigh up the pros and cons of social consequences is likely to contribute to reactive and less sophisticated forms of instrumental violence. Also it is noteworthy that mankind’s ability to reason has been credited as the primary factor responsible for the historical decline of violence [46]. The faculty of reason as defined by our knowledge of the world and our ability to use this knowledge in the pursuit of goals has allowed mankind to perceive conflict as a problem to be solved, to develop cultural institutions to deter violence, and to think through the social consequences of our actions [46]. Social reasoning from this perspective is in part social knowledge, innate social cognitive ability, and also acquired skill. The distinction between instrumental and reactive violence may also help account for some of the discrepancies observed in the literature regarding the relationship between cognition and violence. For instance, Naudts and Hodgins [3] found that people with schizophrenia who have a long history of aggressive behaviour have better executive functioning than those who become violent after illness onset. But the study failed to make a distinction between instrumental and reactive violence and it may be that those with long histories of aggressive behaviour were primarily committing instrumental acts of instrumental violence thus requiring higher levels of executive functioning.

### Paradigms for measuring violence in schizophrenia

There is much to recommend the study of inpatient violence for the purpose of disentangling the relationship between neurocognition and violence. The accurate measurement of violence in the community is beset by several methodological challenges such as reliance on self-report, or information being documented in police files concerning arrest or conviction. All of these may be incomplete. Violence in the community however is likely to be a more realistic test of risk assessment and prediction. In contrast, measures of staff-observed inpatient violence are likely to be more objective and complete, though the number of actual incidents of violence is likely to be reduced by intensive nursing care and de-escalation. Both inpatient and outpatient violence occur in instrumental and reactive varieties. Also meta-analytic reviews have found that the strength and direction of violence risk factors are the same for inpatient and outpatient violence [1, 14, 15]. To date only a few inpatient prospective studies have been carried out to explore the relationship between neurocognitive deficits and violence [47–49]. All of these studies have found a positive relationship in samples of patients with schizophrenia. None of these studies examined neurocognitive deficits as a distal risk factor for violence or ‘root cause’ whose effect is mediated through more proximal risk factors such as social cognitive deficits, psychiatric symptoms, day to day social functioning and violence risk. Similarly no study has focused on emotional and social reasoning whilst controlling for other risk factors.

### Aims

We hypothesised that for forensic patients with schizophrenia or schizoaffective disorder that a) neurocognitive and social cognitive deficits would be determinants of violence and b) that the relationship between neurocognitive deficits and violence would be mediated by risk factors such as deficits in social reasoning, increased symptoms, impaired social functioning and increased violence proneness.

## Method

### Study design

This is a naturalistic 12 month prospective observational cohort study of cognitive ability (neurocognition and social cognition) as a determinant of violence amongst patients with schizophrenia and schizoaffective disorder in a forensic hospital. Data were gathered from 2012–2013. All assessments for each individual were completed on average over a one month time period. Patients were followed up from the point of assessment for 12 months or until discharge to observe if they had been involved in a violent incident. The assessment consisted of the MATRICS Consensus Cognitive Battery (MCCB) an assessment of neurocogniton and social cognition [22], The Social and Occupational Functioning Assessment Scale (SOFAS) [50], an assessment of ‘real world’ social functioning and the Positive and Negative Symptom Scale (PANSS) [51] an assessment of symptom severity. The Historical Clinical and Risk 20 (HCR-20) was used as an assessment of violence proneness or ‘risk’ [10–12]. Each of these domains was assessed by researchers who were blind to the results of the other assessments. Several patients who consented and participated in the cognitive assessment refused to take part in an assessment of symptoms.

### Participants and setting

The study was approved by the National Forensic Mental Health Service Research and Audit Ethics and Effectiveness committee. All participants gave written informed consent.

The National Forensic Mental Health Service for Ireland provides specialised care for adults who have a mental disorder and are at risk of harming themselves or others. All patients are detained under forensic mental health legislation or special parts of the Mental Health Act, or are conditionally discharged to supervised community places under forensic mental health legislation. At the time of the study the National Forensic Mental Health Service (NFMHS) for Ireland had 94 secure inpatient beds at high, medium and low levels of therapeutic security [52] located on a single campus, the Central Mental Hospital (CMH), and 13 supervised community beds for those discharged subject to conditions [53]. The CMH is the only secure forensic psychiatric hospital for the Republic of Ireland, a population of 4.6 million.

In total 123 patients were deemed eligible to participate during the recruitment phase. Of these, 8 patients declined to take part, 9 were discharged before they could complete the assessment, 1 patient was judged to be feigning during the assessment, and 1 patient did not complete the cognitive assessment.

All participants were diagnosed independently of other assessments by a consultant forensic psychiatrist using the Structured Clinical Interview for DSM-IV-TR [54]. Participants were selected if they met DSM-IV-TR criteria for schizophrenia or schizoaffective disorder. A total of 89 participants (76 with schizophrenia, 13 with schizoaffective disorder) met the inclusion criteria and consented to participate in the study. A further 15 with other diagnoses were excluded. Of the 89 participants, 8 were being supervised in the community for part of the follow-up period and 81 were hospital in-patients throughout.

Five (5.6 %) of the 89 were female. The average age of the 89 patients who participated in the study was 40 years. The mean length of stay was 7.5 years (SD 9.5), median 4.7 years, and mode 5.2 years.

### Cognitive assessment

Patients were assessed using the Measurement and Treatment Research to Improve Cognition in Schizophrenia (MATRICS) Consensus assessment battery of cognitive deficits in schizophrenia [25], and also the Test of Premorbid Functioning TOPF-UK [55]. These assessments were carried out at the same time by masters’ level Assistant Psychologists.

The MATRICS battery covers seven cognitive domains: Processing speed; Attention/ vigilance; Working memory; Verbal learning; Visual learning; Reasoning and problem solving; Social Cognition assessed using social reasoning tasks for managing emotions taken from the Mayer-Salovey-Caruso Emotional Intelligence Test (MSCEIT) [56, 57]. The Managing Emotions subtest of the MSCEIT is a social reasoning test. The test comprises of vignettes of various situations, specified goals, and options for coping with the emotions and social situations depicted in these vignettes. Participants are required to indicate the effectiveness of each solution ranging from one (very ineffective) to five (very effective). We will refer to the sub-test of the MSCEIT used within the MCCB throughout this paper as a measure of social cognition, while acknowledging that there are other measures and other constructs. In validation studies, and in antipsychotic trials of stable patients, the MATRICS demonstrated excellent reliability, minimal practice effects and significant correlations with measures of functional capacity with test-retest reliability of 0.9 for the overall composite score in the original validation study [57]. This value has been consistently found in multisite clinical trials. For example, the reliability was 0.88 in the 29-site study mentioned above [58].

There is evidence that the six neurocognitive sub-scales of the MATRICS can be expressed as three factors [59] but only by excluding the MSCEIT social cognition sub-scale, with an associated loss of sensitivity to social function [59]. Fett et al. [29] have found in a meta-analysis that social cognition is more closely related to social outcomes than is neurocognition. There is also a growing awareness that non-social and social cognition are separable dimensions. Therefore the MCCB scoring system now provides an option for a neurocognitive composite that does not include the social cognition sub-scale [60]. We believe it shows greater fidelity to the design of the MATRICS to first analyse all sub-scales including the social cognition scale separately, and to give the results also for the MATRICS composite score. We have therefore presented results for all seven subscales, and we have combined the six neurocognitive sub-scales into a single neurocognitive composite scale. To analyse neurocognition separately from social cognition a composite neurocognition score was calculated from the mean t-score for the first six items of the MATRICS battery (excluding social cognition) not correcting for age, gender, and education. This method of calculating a composite measure of neurocognition without being contaminated by the social cognitive domain has been widely used within the literature [61].

Scores for estimated pre-morbid intelligence (TOPF-UK) were not adjusted for education as an estimate of premorbid ability because the symptoms associated with mental disorder can affect educational attainment. A small number of patients (12 of 89) could not complete the TOPF-UK because of literacy problems. The mean estimated premorbid IQ was 96.

### Functional performance

The SOFAS [50] was completed by a member of the multidisciplinary team responsible for the care of the patient, who was blind to the other assessments including the cognitive assessment. Functioning assessments were obtained for 86 of the 89 participants.

### Symptom assessment

A PANSS [51] assessment was completed on 77 of the 89 patients. The PANSS assessments were completed independently of the cognitive assessments by a psychiatric registrar and an assistant psychologist trained in its use. The PANSS is designed to be scored for positive, negative and general symptoms, and a total symptom score. Because symptoms may overlap with personality traits relevant to violence such as impulse control, affect regulation, narcissism, and paranoid cognitive personality style [62], the total symptom score may be as good or better a predictor of violence than the positive symptom score alone.

### Assessment of violence risk and need for therapeutic security

The HCR-20 [10], a measure of risk of violence was assessed by forensic psychiatry higher trainees (equivalent to US fellow) who were blind to the other assessments (MD and ZA). The HCR-20 is amongst the most extensively validated risk assessment schemes for use within forensic mental health settings [11]. The historical scale contains ten ‘static’ items: previous violence, young age at first violent incident, relationship instability, employment problems, substance misuse problems, history of major mental illness, psychopathy, childhood maladjustment, personality disorder, and prior supervision failure. The psychopathy item was omitted because it is not routinely assessed. The clinical scale contains five ‘current’ items sensitive to change including lack of insight, negative attitudes, active symptoms of major mental illness, impulsivity and unresponsiveness to treatment. The risk scale contains five ‘future’ items: plans lack feasibility, exposure to destabilisers, lack of personal support, noncompliance with remediation attempts and stress. All items are given equal weight [12]. We have previously described the extent to which the HCR-20 and its individual items when measured at baseline do or do not predict subsequent violence in this population [13]. In the present study the HCR-20 is taken as the means of controlling for violence proneness at baseline.

The DUNDRUM-1 triage security instrument [63] is a static assessment of the need for therapeutic security. It is used as a means of comparing the patients in this forensic hospital with those in forensic hospitals elsewhere. The DUNDRUM-1 triage security instrument includes eleven items rating the seriousness of violence, need for specialist treatments and other indicators of need for high, medium or low levels of therapeutic security. A mean item score of between 3 and 4 indicates a need for high security, between 2 and 3 for medium security, 2 for low security, 1 for open hospital or community settings [64]. Item 1 rates the severity of the most serious violent act, ranging from 0 for none to 4 for fatal or potentially fatal violence.

### Assessment of violence

A psychiatric trainee (EW) who was blind to the scores on other assessments reviewed the incident report forms, patient’s clinical notes and legal forms recording incidents of restraint or seclusion, as well as a separate log of incidents kept in the nursing operational management office. This process identified all violent incidents from multiple cross-referenced sources, following the assessments up to the date of discharge or twelve months follow-up. The 8 patients in supervised community residences for part of the follow-up period were monitored in the same way. An individual was classified as violent if they were the clear instigator or co-aggressor, and if the incident involved harm to staff or other patients. The first violent incident was taken as a means of defining violence as a binary outcome. This outcome measure lends itself to both the receiver operating characteristic (ROC) area under the curve analysis (AUC) and to binary logistic regression and so this has become the recommended way of studying factors predicting violence and other discrete outcomes [11, 65]. Very few patients were violent more than once in the follow-up period so that frequency of violence can be studied only in very large samples.

Violence was further classified into reactive and instrumental violence using Woodward and Porter’s coding scheme [42].

### Medication

A chlorpromazine equivalent (CPZeq) was calculated for each participant as a measure of his/her relative daily dose of antipsychotic medications [66–68].

### Data analysis

All data were analysed using SPSS-22 [69]. Demographics and differences between violent and nonviolent groups are presented in Table 1.

**Table 1:** Mean (SD) comparisons between violent and non-violent groups after controlling for age and gender as co-variants. Effect sizes and AUC for receiver operator characteristics (ROC)

	ANOVA							Effect size			Receiver operating characteristic		
	Non-violent n = 79		Violent n = 10		F-statistic (df 1,87)	P value	Partial Eta squared		95 % CI			95 % CI	
	mean	S.D.	mean	S.D.				d	lower	upper	AUC	lower	upper
Age	40.9	12.7	36.1	9.4	1.342	0.250	-	0.38	−0.27	1.04	0.62	0.45	0.80
Length of stay (years)	8.0	9.7	3.0	5.0	2.243	0.13	-	0.50	−0.16	1.16	0.73	0.54	0.91
Chlorpromazine equivalents	538	366	772	397	3.6	0.063	-	0.64	−0.03	1.30	0.67	0.46	0.88
Pre-morbid IQ (TOPF-UK)	96.0	12.6	96.8	8.1	0.023	0.879	-	0.06	−0.72	0.84	.49	0.31	0.67
PANSS total	62.5	20.0	90.1	19.4	12.2	0.001	.157	1.38	0.58	2.19	0.84	0.71	0.97
PANSS positive	13.7	7.0	21.6	8.7	9.32	0.003	.113	1.21	0.41	2.01	0.79	0.65	0.93
PANSS negative	18.9	7.9	25.0	6.5	6.175	0.111	.078	0.98	0.19	1.78	0.71	0.55	0.87
PANSS general	29.02	10.3	43.6	10.4	14.202	.000	.163	1.49	0.68	2.31	0.86	0.73	0.99
HCR-20 total score	20.8	5.7	28.2	8.0	14.04	0.000	.142	1.26	0.57	1.94	0.78	0.59	0.96
HCR-20 Historical	12.9	2.7	15.2	4.5	5.178	.025	.057	0.763	0.09	1.43	0.75	0.55	0.95
HCR-20 Current	4.50	2.63	7.2	2.78	12.59	.001	.129	1.91	0.51	1.87	0.76	0.59	0.94
HCR-20 Risk	3.39	2.12	5.80	2.29	10.06	.002	.106	1.064	0.39	1.74	0.77	0.62	0.71
SOFAS	59.2	17.2	35.6	18.9	14.9	0.001	-	1.29	0.61	1.98	0.83	0.66	0.99
DUNDRUM-1 (11 item)	29.54	5.01	27.40	7.1	.994	0.322	.012	0.334	−0.33	0.99	0.44	0.21	0.66
DUNDRUM-1 (9 item)	27.08	3.93	23.90	6.93	3.275	0.074	.037	0.607	−0.06	1.27	0.38	0.16	0.60

To correct for multiple hypothesis testing for the seven cognitive domains comprising the MATRICS battery group differences across all subtests and the neurocognitive and MATRICS composites were analysed using multivariate analysis of variance, with age and gender entered as co-variates. Group differences across cognitive domains and composite scores were analysed using one way ANOVAs. Bonferroni correction was applied as a conservative check on multiple hypothesis testing. Similarly for the PANSS and HCR-20 all subscales including the total scales were analysed using multivariate analysis of variance, with age and gender as co-variates.

The ability of baseline measures to discriminate those who during the follow-up period committed violent incidents was analysed using the receiver operating characteristic (ROC) area under the curve (AUC). An association was deemed significant if the lower limit of the 95 % confidence interval of the AUC was greater than 0.5, the line of random information.

Correlations were calculated using Spearman’s non-parametric method as violence is a binary variable.

**Fig. 1:**
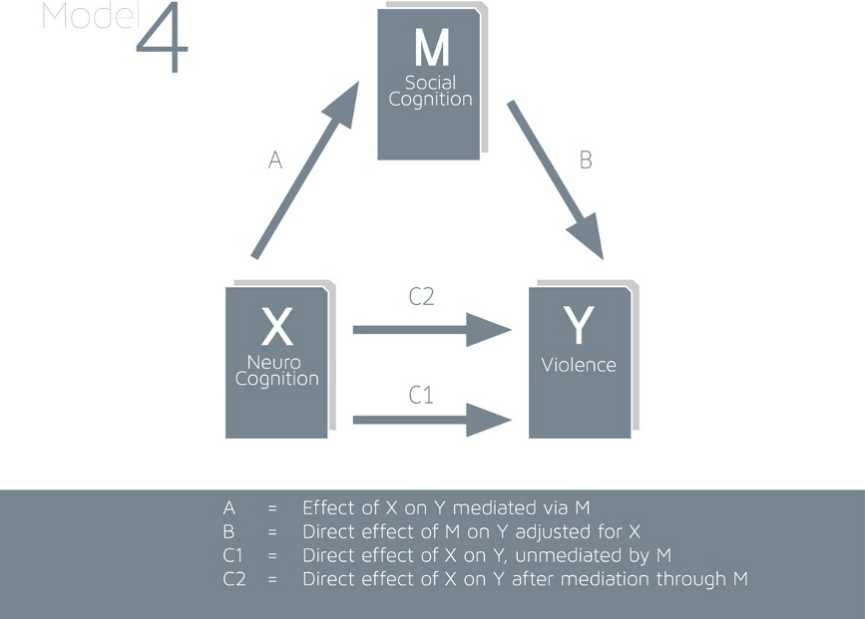
Mediation model 4: single mediator as in Table 4

SPSS PROCESS macro model 4 [70] was used to analyse mediation relationships between antecedent factors such as neurocognition, social cognition, and the dichotomous outcome violence (Fig. 1). Age and gender were entered as co-variants in all mediation analysis. SPSS PROCESS macro is a computational tool for path analysis-based moderation and mediation analysis. Various measures of effect size for indirect effects are generated in mediation models. Effect sizes were calculated as regression coefficients in the first instance and later as odds ratios to facilitate interpretation. Bootstrapping was used to estimate indirect effects, and 95 % bias-corrected confidence intervals were used for the indirect effects using 1,000 bootstrap samples. A confidence interval for an odds ratio that does not contain a score of one indicates statistically significant mediation.

Mediation effects were in each case examined for all combinations to determine the direction of the causal effect. If a relationship between an antecedent factor, a mediating factor and violence does not hold true when the order of antecedent and mediating factors is switched this has been taken as support for preferring one pathway (an ordering of factors) over another.

**Fig. 2:**
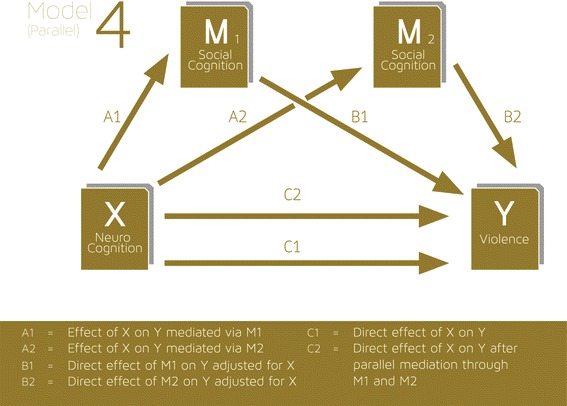
Mediation model 4: two or more mediators, parallel model

**Fig. 3:**
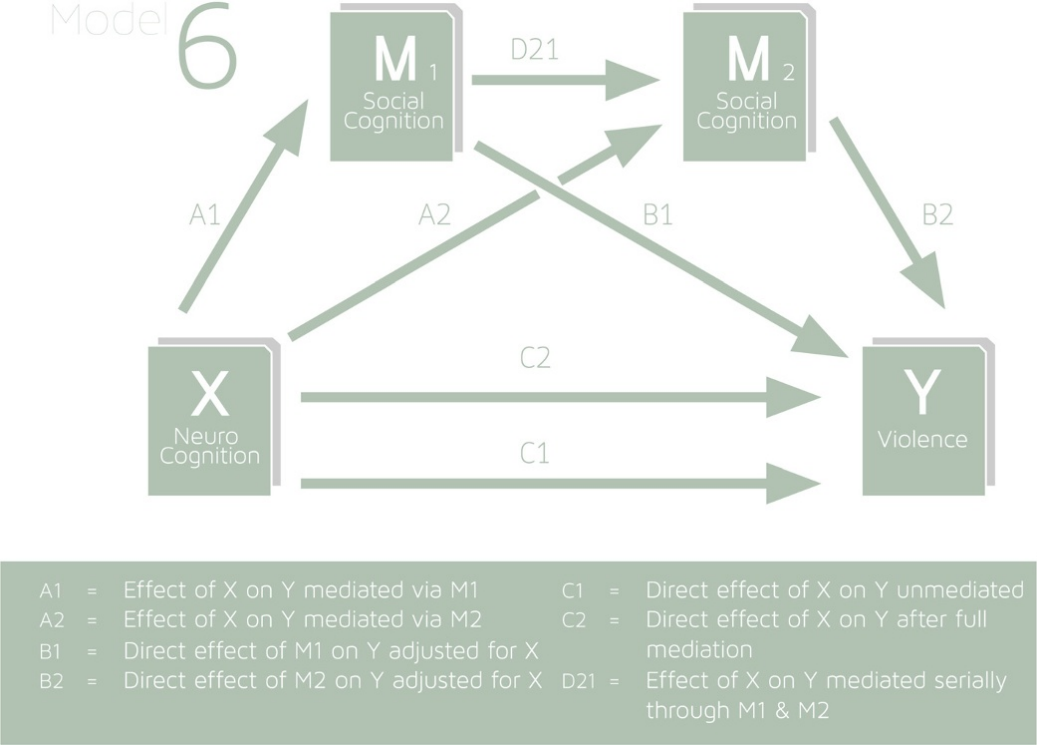
Mediation model 6: two or more mediators, serial mediation

We also tested more complex mediation models involving two or more mediators employed SPSS PROCESS macro models 4 (parallel) (Fig. 2) and model 6 (Fig. 3) (serial) [70]. These models were regarded as exploratory.

## Results

The mean follow-up period (*n* = 89) was 1.22 years (SD 0.44). There were 107.4 person-years at risk. During the follow-up period, 10 of the 89 patients with schizophrenia-schizoaffective disorders committed violent acts (base rate 9.7/100 person-years at risk). Note that only the first violent incident for each person was counted. All violent incidents were coded [42] as reactive violence, with two rated as also having a minor instrumental element. On the DUNDRUM-1 item 1 measure of seriousness of violence (scored 0 to 4 where ‘4’ is fatal or life threatening) eight violent incidents were rated ‘2’ and the remaining two were rated ‘1’.

A relationship between gender and violence did not reach statistical significance, as 2/10 who were violent were female, compared to 3/79 who were not violent, Fisher’s exact test = 4.39, p = 0.095.

All of the participants had a history of past violence as recorded by the HCR-20 and DUNDRUM-1 triage security instrument. On item 1 of the DUNDRUM-1 triage security instrument, 62 patients scored ‘4’, indicating a history of homicide or life threatening violence to others and 20 scored ‘3’ indicating other serious violence. On the HCR-20 item 1. 86 scored ‘2’ indicating a history of serious or repetitive violence to others.

The mean score on the DUNDRUM-1 eleven item scale was 29.5 (SD 5.0) and for the DUNDRUM-1 nine item scale omitting self-harm items, the mean score was 27.1 (SD 3.9), a mean score per item of 3.0 (SD 0.4). The mean for the Total HCR-20 In was 20.8 (SD 5.7), median 21.0, mode 17.

The mean t-score of the MATRICS composite score for all patients was 17.9 (SD 13.2, range −11.0 to 51.0). The published population norm is a t-score of 50 (SD 10). This group of forensic hospital patients with schizophrenia is therefore more than three standard deviations below the population norm. Table 2 shows that for the group who were not violent during follow-up the MATRICS composite represented as a mean t-score was 20.9 (SD 14.0). The violent group was even more impaired (12.8, SD 9.1).

**Table 2:** Mean (SD) comparisons for t-scores on MATRICS domains and composites, comparing violent and non-violent groups after controlling for age and gender as co-variants. Effect sizes and AUC for receiver operator characteristics (ROC)

MATRICS Domains and Composites	ANOVA							Effect Size d			Receiver operating characteristic		
	Non-violent n = 79		Violent n = 10		F-statistic	P value	Partial Eta squared	d	95 % CI		AUC	95 % CI	
	Mean	S.D	mean	S.D	(df 1,87)				lower	upper		lower	upper
Processing Speed	24.82	15.5	18.50	10.2	5.242	0.025	0.058	0.76	0.10	1.43	0.65	0.51	0.79
Attention	28.4	11.1	23.8	10.4	2.43	0.122	0.028	0.52	−0.13	1.18	0.62	0.43	0.81
Working Memory	31.3	12.7	32.8	8.4	0.053	0.818	0.001	0.24	−0.41	0.90	0.45	0.30	0.60
Verbal Learning	33.9	7.6	28.4	4.5	7.56	0.007*	0.082	0.92	0.25	1.59	0.72	0.58	0.86
Visual Learning	32.7	12.6	26.5	11.9	2.57	0.11	0.029	0.53	−0.12	1.20	0.64	0.46	0.82
Reasoning	35.9	7.4	35	5.4	0.808	0.371	0.009	0.30	−0.35	0.96	0.51	0.33	0.68
Social Cognition	35.7	11.0	24.4	6.3	11.57	0.001*	0.12	1.14	0.46	1.82	0.81	0.71	0.90
Neuro-cognitive composite	31.1	9.3	27.5	6.7	3.05	0.084	0.035	0.58	−0.07	1.24	0.61	0.45	0.76
MATRICS Total Composite	20.9	14.0	12.8	9.1	5.720	0.019	0.063	0.80	0.13	1.47	0.67	0.53	0.81

*Is significant following Bonferroni Correction

### Differences in cognitive ability between violent and nonviolent groups

One-way MANOVA showed that violent patients had significantly worse neurocognitive and social cognitive abilities than non-violent patients (Pillai’s Trace V = 0.339, F (8, 78) = 5.008, *p* < 0.001, Partial Eta squared = 0.339) after controlling for age and gender.

Violent patients performed significantly worse than non-violent patients on the MATRICS domains of processing speed, verbal learning, social cognition and the MATRICS total composite (Table 2). Following Bonferroni correction for multiple testing the violent and non-violent groups differed only on the verbal learning domain and the social cognitive domain. The magnitudes of the differences between violent and nonviolent groups are also presented as effect sizes (Cohen’s d) in Tables 1 and 2.

For PANSS scores, one-way MANOVA showed that violent patients had significantly higher levels of psychopathology (Pillai’s Trace V =0.172, F (4, 70) = 3.639, *p* < 0.009, Partial Eta squared −0.172) (Table 1).

One-way MANOVA showed that HCR-20 total scores for risk of violence were higher for violent patients (Pillai’s Trace V =0.149, F (3, 83) = 4.839, *p* < .004, Partial Eta squared −0.149) (Table 1).

### Predicting violence

Three of the seven neurocognitive domains of the MATRICS - processing speed, verbal learning, and social cognition had AUCs significantly greater than random. The MATRICS composite was also significantly better than random (Table 2). The social cognitive domain of the MATRICS had the highest AUC. Although the MATRICS composite was predictive of violence the Neurocognitive composite without the addition of the Social Cognitive Domain was not.

The total HCR-20 score, PANSS positive, PANSS negative, PANSS general and PANSS total scores all had ROC AUC scores that were significantly better than random.

### Correlations between cognition, real world functioning, violence risk and violence

Table 3 depicts non-parametric Spearman correlations between cognition (both neurocognition and social cognition), social functioning using the SOFAS, proneness to violence (risk of violence) using the HCR-20 total score, past history of homicide or lethal violence (DUNDRUM-1 item 1) and actual violence during the follow-up period. These can be summarised as showing that social cognition and neurocognition correlated positively with each other and with social function (SOFAS). They correlated negatively with symptom severity (PANSS total), violence proneness (HCR-20 total score), and subsequent actual violent acts. It is notable that neurocognition did not correlate directly with PANSS positive symptoms, though it did correlate negatively with PANSS negative symptoms and PANSS general symptoms. Social cognition (MSCEIT/MATRICS) tended to have the strongest correlations with all symptom measures and with subsequent violence, while neurocognition had stronger correlations with the HCR-20 and SOFAS scores. An incidental finding was that less impaired social cognition was associated with a past history of lethal or life-threatening violence (a score of ‘4’ on DUNDRUM-1 item 1).

**Table 3:** Spearman correlations. Each column is divided into three cells per row. These are the Spearman correlation coefficient, p value and number of subjects for each row

		1	2	3	4	5	6	7	8	9	10	11
1	Social Cognition	-										
2	Neuro-cognition Composite	0.397	-									
		.001										
		89										
3	MATRICS Composite (includes neuro-cognition and social cognition)	0.541	0.984	-								
		.001	.001									
		89	89									
4	PANSS Total	−0.461	−0.338	−0.405	-							
		.001	.003	.001								
		77	77	77								
5	PANSS Positive	−0.361	−0.149	−0.217	0.773	-						
		.007	.195	.058	.000							
		77	77	77	77							
6	PANSS Negative	−0.398	−0.359	−0.406	0.729	0.323	-					
		.001	.001	.001	.001	.004						
		77	77	77	77	77						
7	PANSS General	−0.473	−0.298	−0.371	0.917	0.735	0.571	-				
		.001	.009	.001	.001	.001	.001					
		77	77	77	77	77	77					
8	HCR-20 Total In	−0.252	−0.314	−0.343	−0.666	0.567	0.543	0.614	-			
		.017	.003	.001	.001	.001	.001	.001				
		89	89	89	77	77	77	77				
9	SOFAS	0.411	0.521	0.556	−0.617	−0.438	−0.499	−0.542	−0.616	-		
		.001	.001	.001	.001	.001	.001	.001	.001			
		86	86	86	77	77	77	77	86			
10	History of Homicide or lethal violence D1 item 1	0.254	−0.021	0.028	−0.210	−0.145	−0.197	−0.092	−0.215	0.082	-	
		.016	.848	.759	.067	.207	.087	.425	.043	.453		
		89	89	89	77	77	77	77	89	86		
11	Violence	−0.340	−0.122	−0.194	0.343	0.293	0.214	0.362	0.308	−0.351	−0.288	-
		.001	.255	.069	.002	.010	.062	.001	.003	.001	.006	
		89	89	89	77	77	77	77	89	86	89	

### Mediation between neurocognition, social cognition and violence

The relationship between neurocognition and violence was completely mediated by the social cognitive domain of the MATRICS, after co-varying for age and gender (Table 4).

**Table 4:** In all cases, the outcome (Y) is ‘violent act’. X is the hypothesised determinant factor and M is the hypothesised mediating factor. See also Additional file 1 for figures representing these effects and pathways

	C1: Direct effect X on Y before mediation			C2: Direct effect X on Y after mediation			A: Mediated effect X on Y via M			B: Direct effect M on Y adjusted for X		
M	OR	95 % CI		OR	95 % CI		OR	95 % CI		OR	95 % CI	
		lower	Upper		lower	Upper		lower	upper		Lower	upper
X = Neurocognitive composite	0.929	0.850	1.016									
social cognition				0.989	0.893	1.095	0.932	***0.849***	***0.976***	0.869	***0.779***	***0.970***
PANSS				0.955	0.836	1.092	0.947	***0.821***	***0.994***	1.069	***1.016***	***1.125***
SOFAS				1.008	0.904	1.124	0.916	**0.788**	**0.997**	0.931	***0.882***	***0.982***
HCR-20				0.972	0.881	1.072	0.950	***0.831***	***0.997***	1.214	***1.058***	***1.393***
X = social cognition	0.864	**0.781**	**0.957**									
neurocognition				0.869	***0.779***	***0.970***	0.997	0.934	1.031	0.989	0.893	1.095
PANSS				0.886	***0.774***	***0.986***	0.958	0.791	1.034	1.054	0.997	1.114
SOFAS				0.906	0.811	1.012	0.964	0.817	1.052	0.953	0.901	1.007
HCR-20				0.890	***0.803***	***0.987***	0.973	0.870	1.065	1.187	***1.025***	***1.374***
X = symptoms (PANSS total score)	1.067	**1.017**	**1.120**									
neurocognition				1.069	***1.015***	***1.126***	1.006	0.985	1.126	0.955	0.836	1.092
Social cognition				1.054	0.997	1.114	1.029	0.995	1.107	0.886	0.774	1.014
SOFAS				1.042	0.986	1.102	1.037	0.909	1.136	0.935	0.867	1.009
HCR-20				1.032	0.969	1.099	1.043	0.915	1.165	1.170	0.953	1.436
X = social function (SOFAS)	0.931	**0.887**	**0.978**									
Neurocognition				0.930	***0.881***	***0.982***	1.002	0.958	1.046	1.008	0.904	1.124
Social cognition				0.953	0.901	1.007	0.973	0.913	1.005	0.906	0.811	1.012
PANSS				0.935	0.867	1.009	0.972	0.866	1.092	1.042	0.986	2.633
HCR-20				0.971	0.920	1.024	0.942	0.753	1.008	1.320	***1.095***	***1.614***
X = violence proneness (HCR-20 total score)	1.228	**1.075**	**1.403**									
Neurocognition				1.214	***1.058***	***1.393***	1.012	0.956	1.085	0.972	0.881	1.072
Social cognition				1.187	***1.027***	***1.371***	1.055	**1.000**	**1.166**	0.890	***0.797***	***0.987***
PANSS				1.170	0.953	1.436	1.102	0.817	2.175	1.032	0.984	1.107
SOFAS				1.320	***1.088***	***1.601***	1.058	0.759	1.392	0.971	0.917	1.022

Age and Gender as covariates. C1, C2, A and B refer to the labelling in Fig. 1. Confidence intervals underlined and in bold are significant

Figure 1 shows the mediation model in schematic form. Table 4 shows these effects expressed as odds ratios.

Neurocognition appears to have no influence on violence independent of its effect on social cognition (Table 4). There was no evidence that neurocognition mediated the relationship between social cognition and violence. In total the effect of neurocognition, social cognition, and age and gender could account for 35 % (Nagelkerke R^2^) of the variance in incidence of violence.

### PANSS Total score as a mediator between neuro-cognition and violence

The PANSS total score completely mediated the relationship between neurocognition and violence. The indirect effect of neurocognition on violence as mediated by the PANSS total score was OR = 0.94. There was no evidence that neurocognition mediated the relationship between psychiatric symptoms (PANSS total) and violence (Table 4). In total the effect of neurocognition, symptoms, and age and gender could account for 48 % (Nagelkerke R^2^) of the variance in the incidence of violence.

### Social functioning as a mediator between neurocognition and violence

Social functioning (SOFAS) completely mediated the relationship between neurocognition and violence after controlling for age and gender. The indirect effect of Neurocognition on Violence as mediated by the SOFAS score was OR = 0.91. There was no evidence that neurocognition mediated the relationship between social functioning and violence. In total the effect of neurocognition, social functioning, and age and gender could account for 34 % (Nagelkerke R^2^) of the variance of violent incidents.

### HCR-20 Violence risk as a mediator between neuro-cognition and violence

Violence proneness (risk of violence) as measured by the HCR-20 total score completely mediated the relationship between neurocognition and violence. The indirect effect of neurocognition on violence as mediated by the HCR-20 total was OR = 0.95. There was no evidence that neurocognition mediated the relationship between violence risk and violence. In total the effect of neurocognition, HCR-20, and age and gender could account for 35 % (Nagelkerke R^2^) of the variance of violent incidences.

### Neurocognition as the foundation for the emergence of violence risk factors

In addition to the consistent evidence of mediation between neurocognition and violence (Table 4), there was evidence that the relationship between social cognition and violence was mediated in part by social functioning (SOFAS), and the relationship between social functioning (SOFAS) and violence was mediated in part by violence proneness (HCR-20 violence risk). To test the hypothesis that neurocognitive impairments represent the foundation for the emergence of a range of risk factors for violence such as social cognitive deficits, increased symptoms, impaired functioning and HCR-20 violence risk we constructed a serial mediation model (Fig. 3, model 6 of the PROCESS macro [70]). When all four mediating factors were entered into a serial mediation model between neurogonition and violence, there was no evidence of serial mediation from neurocognition, to social cognition, to psychiatric symptoms, to social functioning, to HCR-20 violence proneness. Nor was there evidence of serial mediation between any three of the four mediating variables. Also there no evidence of serial mediation between any two of the four mediating variables.

When all four mediating variables are entered into a parallel mediation model (Fig. 2, model 4 of the PROCESS macro [70]) there is no evidence of an indirect mediated effect between neurocognition and violence. When every combination of three out of the four mediating variables is entered into the parallel mediation model (Model 4) there was again no evidence for an indirect mediated effect between neurocogonition and violence. When each possible pair of the four mediating variables was entered in the parallel model (model 4) there was evidence that the total indirect effect between neurocognition and violence was significant, completely mediated by social cognition and HCR-20 violence risk as two parallel pathways from neurocognition to violence (total indirect effect expressed as odds ratio 0.896, 95 % CI 0.730 – 0.971). Altogether this model could account for 46 % of the variance of violent incidents. There were no other robust effects mediated by any other pair of mediating factors.

### Social cognition and symptoms as a mediator between neurocognition and violence

Although psychiatric symptoms did not mediate the relationship between social cognition and violence (Table 4), because of the link between delusions and violence [1, 14, 15] and the association between social cognition and symptoms (Table 3), we wanted to investigate whether there would be evidence of serial mediation between neurocognition and violence when social cognition and symptoms were added to the model (Process Macros Model 6). We omitted the measure of violence proneness or risk (HCR-20) because of likely overlap in content between some items in the HCR-20 and the measure of symptom severity (PANSS). As set out above, there was no evidence that social cognition and symptoms mediated the relationship between neurocogntion and violence, either serially or in parallel.

## Discussion

### Main findings

In this prospective cohort study of forensic hospital patients with schizophrenia and schizoaffective disorder we found a robust association between cognitive (neurocognitive and social cognitive) deficits and violence. Using multivariate analysis the cognitive domains measured by the MCCB could account for 34 % of the variance in violent incidents after controlling for age and gender during a 12 month follow up. Both nonviolent and violent patients had significant impairments in neurocognition and social cognition. The mean MCCB composite was three standard deviations below a nonclinical mean. Also even though these forensic patients were admitted because of a prior history of violence, most were not violent during the period of study. Of all the MCCB domains, performance on the social reasoning test (MSCEIT) produced the largest effect size.

When the influence of neurocognition on violence was explored using mediation analysis, neurocognition emerged as a distal risk factor whose effect on violence occurred through more proximal risk factors. The relationship between neurocognition and violence was completely mediated by social cognition (MSCEIT), violence proneness (HCR-20 Total), psychiatric symptoms (PANSS total), and social functioning (SOFAS). There was also evidence of parallel mediation from neurocognition through social cognition and through violence proneness (violence risk, HCR-20) to violence. This may cast some light on why risk factors within the HCR-20 such as employment problems and prior supervision failure that ought to operate mainly in the community, none-the-less remain predictive in hospital. These risk items may be markers of general dysfunction underpinned by cognitive impairment. In contrast to neurocognition, social cognition as measured by a social reasoning task (MSCEIT) had a direct effect on violence even when controlling for violence proneness (HCR-20 Total Score), psychiatric symptoms (PANSS), and neurocognition. The direct effect of social cognition on violence was however attenuated to insignificance by mediation through a measure of general social function (SOFAS).

### Differences between violent and nonviolent group during 12 month follow up

The greatest difference between violent and nonviolent groups was on the MATRICS social cognition domain, a social and emotional reasoning task assessing patients’ ability to manage emotions. Significant differences were also observed for the neurocognitive measures of verbal learning and processing speed. There was no significant difference between chlorpromazine equivalents of antipsychotic medication between violent and nonviolent groups. In this prospective study of violent outcomes, social cognition measured at baseline produced ROC AUCs comparable with the HCR-20, one of the most widely used violence risk assessment and management schemes. Impaired emotional and social reasoning ability as measured by the MSCEIT appeared to be a determinant of reactive, impulsive violent behaviour.

### Mediation analysis

These findings were further explored using mediation analysis. There was no evidence that neurocognition had an effect on violence independent of social cognition. The composite measure of neurocognition was only related to violence in so far as it affected social and emotional reasoning. Using this model, neurocognitive difficulties amongst people with schizophrenia spectrum disorders in a forensic hospital did not have a direct effect on violence but neurocognitive problems leading to difficulties with social and emotional reasoning did.

For patients with schizophrenia and schizoaffective disorder the relationship between neurocognition and violence was also completely mediated by symptoms (PANSS total score), by social functioning (SOFAS) and by violence proneness (HCR 20 violence risk). Although neurocognitive impairments are thought to occur before the onset of psychosis and to underpin functional impairment to be sure of the causal direction we tested all possible combinations of factors. There was no evidence that neurocogniton mediated the relationship between any of the described variables and violence. Of all of the variables examined, neurocognition was the only independent variable whose effects on violence consistently showed evidence of mediation. Neurocognition therefore appears to be a distal risk factor for violence whose influence only becomes manifest through more proximal risk factors such as social cognition, symptoms, functioning and the risk factors contained within the HCR-20.

There was a significant indirect effect of neurocognition on violence that was mediated by social cognition and violence proneness (HCR-20) in parallel. This was the only higher order mediation found, though this may reflect the size of the sample. The effect of social cognition on violence was independent of violence proneness and symptoms.

### Strengths

This study contained a number of methodological strengths. First to our knowledge this is the only prospective cohort study of patients with schizophrenia and schizoaffective disorder that has examined the relationship between cognition (neurocognition and social cognition) and violence using the MATRICS Consensus Cognitive Battery (MCCB). The MCCB demonstrated its value within a forensic setting. There was evidence of concurrent validity including large and moderate correlations with independently rated measures of social functioning, psychiatric symptoms and violence proneness (violence risk).

Second, for the most part violence is not a homogenous entity. This difficulty was overcome by using an established coding scheme for classifying instrumental and reactive violence. All violent acts in this study were reactive. Violent acts often contain instrumental and reactive elements and those prone to premeditated or instrumental violence also often act violently on impulse or reactively. However it is less common for those who are mainly prone to reactive violence to be instrumentally violent [43]. The association between cognitive impairment (neurocognition and social cognition) and violence observed in this prospective study is strictly speaking an association with reactive acts of violence. However Table 3 shows a retrospective association between the seriousness of the violence leading to admission to the forensic hospital and the MSCEIT measure of social cognition in the MCCB that is positive, the more socially competent, the more serious was the past violence (Pearson r = 0.246, p = 0.020, *n* = 89). These acts were usually delusionally driven and were not always reactive. There is some evidence for differing developmental origins of schizophrenia that may be associated with different patterns of violence [3, 71, 72]. Clarifying this relationship will require further study.

Third, this study is one of a small number of prospective cohort studies of patients with schizophrenia and schizoaffective disorder evaluating cognitive (neurocognitive and social cognitive) determinants of violence against persons [47–49] and therefore satisfies the temporal and association criteria for causal inference.

### Limitations

This study took place within a secure forensic setting which may limit the generalisability of the findings for non-forensic or community settings or in prisons. However within any setting whether community or forensic, patients with schizophrenia who are at risk of violent behaviour are best identified using a reliable and valid risk assessment instrument. This study assessed violence proneness in forensic patients using the range of violence risk factors captured by the HCR-20 which has been validated in many settings [11].

The patients in this study were predominantly male. It is possible that different processes mediate violence in women patients. It has also been suggested that inpatient and outpatient violence are not comparable and that the structured routine, close observation and proximity to others within inpatient settings may be a determinant of violence. However, more recent research suggests that the risk factors predictive of outpatient violence are also predictive for inpatient violence. A history of substance abuse for example is a robust risk factor for violence amongst psychiatric patients in outpatient settings, but is also a risk factor for violent behaviour within inpatient settings, even where substance abuse prior to violent behaviour can be ruled out [13]. Similarly within forensic settings (hospital and community residences) medication adherence is carefully monitored and controlled but this risk factor remains predictive [13].

Although it was not possible to assess psychiatric symptoms concurrently with violent acts in this study, there were significant baseline differences between violent and nonviolent groups on the PANSS total score. Because the neurocognitive cognitive decline observed amongst patients with schizophrenia is thought to occur before the onset of psychosis [16, 17] as does the impairment in social cognition [27] it would be reasonable to infer that cognition (neurocognition and social cognition) influences symptoms rather than the other way round. However because the PANSS data was assessed at baseline only, it is not possible to be more definitive concerning whether psychiatric symptoms immediately preceded violent incidents. Although mediation effects between neurocognition, social cognition, symptoms, social functioning, violence proneness (risk) and violence worked only one way, causal statements about the relationship between neurocognition, psychiatric symptoms and violent behaviour therefore must be qualified.

We did not find evidence for serial or higher order parallel mediation pathways involving psychiatric symptoms, but this may be due to the size of the cohort. Further studies with larger numbers would be helpful.

### Implications

These results are in keeping with the wider literature suggesting that cognitive difficulties (neurocognitive and social cognitive difficulties) are a risk factor for violence in many diagnostic groups [31–33, 48]. The nature of social cognition is itself a matter for continuing research and debate, although it is already recognised that deficits in social cognition occur in a range of mental disorders including autism and schizophrenia [35]. Recent genetic research has demonstrated an overlap amongst the many single nucleotide polymorphisms for schizophrenia, bi-polar affective disorder, attention deficit hyperactivity disorder and autism [73]. An overlap symptom profile or phenotype has been described for patients with schizophrenia and patients with autism spectrum disorder, consisting of selected symptoms from the PANSS negative and general symptom scales [74] A recent empirical review has shown that the relationship between neurocognition and functioning in schizophrenia is significantly mediated by social cognition so that neurocognition influences social cognition which in turn influences functioning [30, 75]. More specifically the finding that social cognitive difficulties as measured by the MATRICS/MSCEIT were directly related to violence is also in keeping with social cognitive theories of violence and with evolving social reasoning being credited for the historical decline of violence [46].

The indirect influence of neurocognition on violence may also help explain some of the discrepancies observed within the literature; where some studies have found a relationship between cognition and violence whereas others have not. Also although much work has been done identifying risk factors for violence in people with schizophrenia and schizoaffective disorder the relationships amongst risk factors have been scarcely studied. One cross-sectional study has reported that in patients with schizophrenia, mentalisation, defined as the ability to attribute mental states to others, mediates the relation between psychopathy and type of aggression. This mediation is facilitated by a specific mentalising profile characterised by the presence of intact cognitive and deficient emotional mentalising capacities associated with deliberate aggression [76]. Deficits in mentalisation have also been associated with self-reported aggression in cross-sectional studies [77]. The current study sheds light on the relationship between a range of variables and subsequent actual violence.

Research on related constructs such as mentalisation and metacognition may help guide future research on treatment. Mediation analysis may help elucidate the relationship between a range of variables which could be targeted by psychological intervention. Deficits in mentalisation for example may mediate attachment styles and the expression of personality traits or personality clusters [77]. Also although measures of metacognition have not been found to distinguish between forensic and non-forensic patients with schizophrenia [78] metacognition may mediate symptom severity and social dysfunction [79]. Evidence of the relationship between delusions and violence in schizophrenia that is mediated through anger and confirmed by temporal proximity may represent an experimental confirmation of this concept [80, 81]. The relationship between delusions, anger and violence [82, 83] has at times been referred to as ‘affect-logic’ [83–85].

Recently several psychotherapeutic approaches have been developed to improve various neurocognitive and social cognitive domains in schizophrenia including cognitive remediation therapy [86–88], metacognitive approaches [89, 90] and mentalisation- based treatment [90, 91], all of which may prove useful for reducing violence risk for patients with schizophrenia. Improvements in social and emotional reasoning on an ability test such as the MSCEIT may be a useful intermediary marker regarding the effectiveness of these programmes. This study formed part of the preliminary work for a study of cognitive remediation therapy in schizophrenia and schizoaffective disorder. We believe there is now a need for a range of studies of means to improve neurocognition and social cognition in patients with schizophrenia in order to improve social function and reduce risk factors for violence and other adverse outcomes.

The findings of this study may also have implications for understanding mental capacity amongst patients with schizophrenia. The current legal model that distinguishes between dynamic impairments of mental capacity supposedly due to psychiatric symptoms and fixed impairments of mental capacity due to intellectual disability may prove to be a false dichotomy. The legal model assumes that when symptoms of schizophrenia spectrum disorders resolve, general and function specific mental incapacities will also resolve. This may also prove to be a false assumption. However there is some tentative evidence that the metacognitive therapy of Moritz et al. [89] may enhance functional mental capacities relevant to competence and legal status [92].

## Conclusions

Research in schizophrenia should concentrate on functional outcomes. Violence is itself evidence of impaired social function, as well as a cause of stigma. In this study, impairments of neurocognition and social cognition experienced by forensic patients with schizophrenia and schizoaffective disorder accounted for a large portion of the variance of subsequent violent behaviour. However the link is nuanced and indirect. Deficits in social reasoning may be more important than other neurocognitive abilities. Neurocognition appears to be linked to violence insofar as it affects higher level social reasoning processes, psychiatric symptoms, social functioning, and violence proneness as measured by the HCR-20 violence risk scores. The neurocognitive difficulties experienced by forensic patients with schizophrenia and schizoaffective disorder may therefore create the foundation for a range of risk factors and impairments of function, which in turn are causally related to violence.

## Additional file

Additional file 1:
**Mediation effects demonstrated.**

## Abbreviations

HCR-20: Historical-clinical-risk management-20
DSM-5: Diagnostic and statistical manual of mental disorders, 5th edition
ICD-10: International classification of diseases, 10th edition
MATRICS: Measurement and treatment research to improve cognition in schizophrenia
MCCB: MATRICS Consensus cognitive battery
MSCEIT: Mayer-Salovey-Caruso Emotional Intelligence Test
WAIS: Wechsler adult intelligence scale
NART: National adult reading test
SOFAS: The social and occupational functioning assessment scale
PANSS: Positive and negative symptom scale
NFMHS: National forensic mental health service
CMH: Central Mental Hospital
DSM-IV-TR: Diagnostic and statistical manual of mental disorders fourth edition text revision
TOPF-UK: Test of premorbid functioning
DUNDRUM: Dangerousness, understanding, recovery and urgency manual
ROC: Receiver operating characteristic
AUC: Area under the curve
CPZeq: Chlorpromazine equivalents
SPSS-22: Statistical package for the social sciences, version 22
ANOVA: Analysis of variance
SD: Standard deviation
95 % CI: 95 percent confidence interval
MANOVA: Multivariate analysis of variance
OR: Odds ratio
