# Supplementary material for: Prospective cohort study of the relationship between neuro-cognition, social cognition and violence in forensic patients with schizophrenia and schizoaffective disorder
Source: BMC Psychiatry. 2015 Jul 10;15:155. doi: 10.1186/s12888-015-0548-0 (PMC4496853; doi:10.1186/s12888-015-0548-0)
Supplement: Additional file 1: — Mediation effects demonstrated. [file 12888_2015_548_MOESM1_ESM.pptx]

## Slide 1
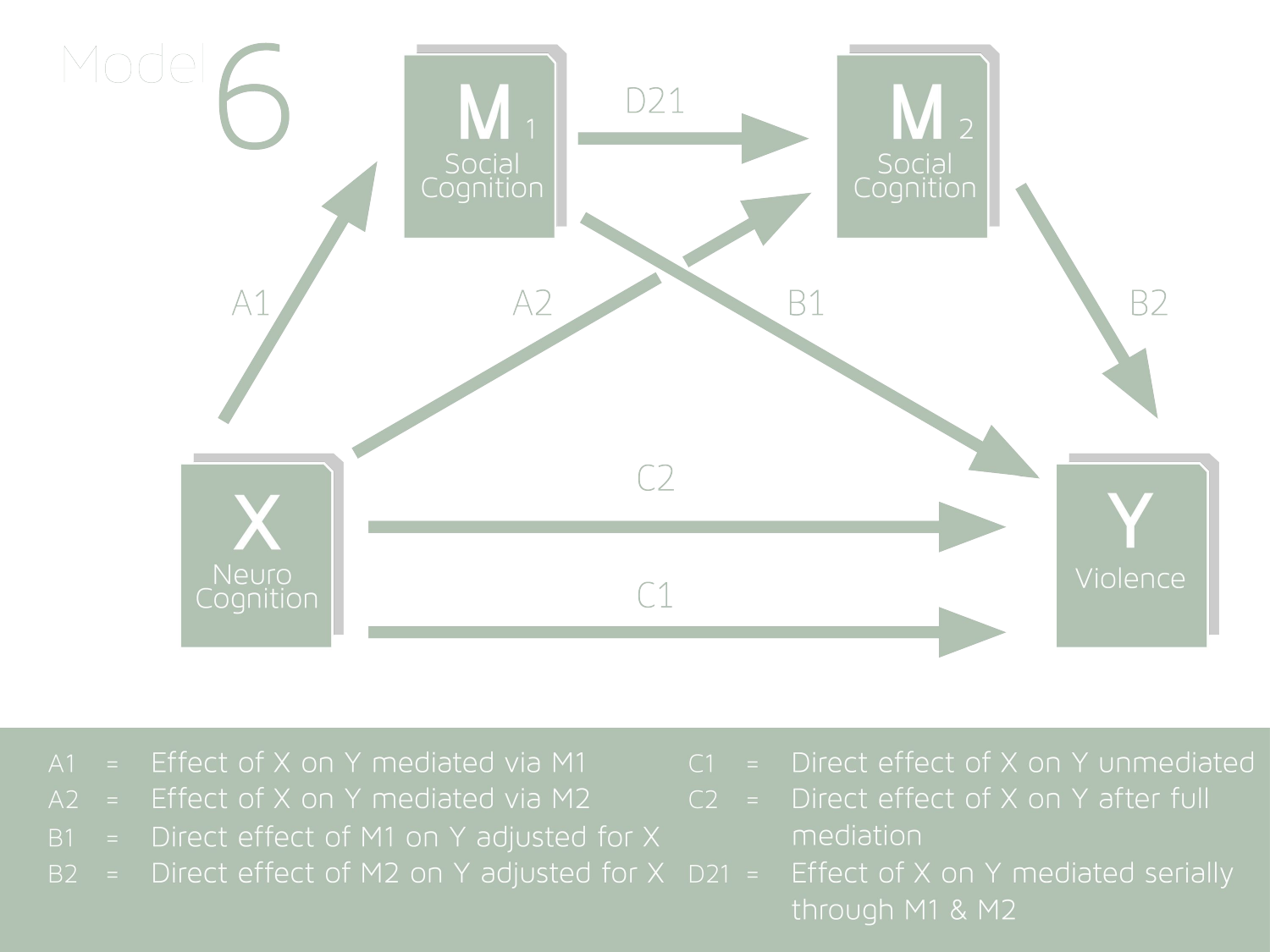

#

## Slide 2
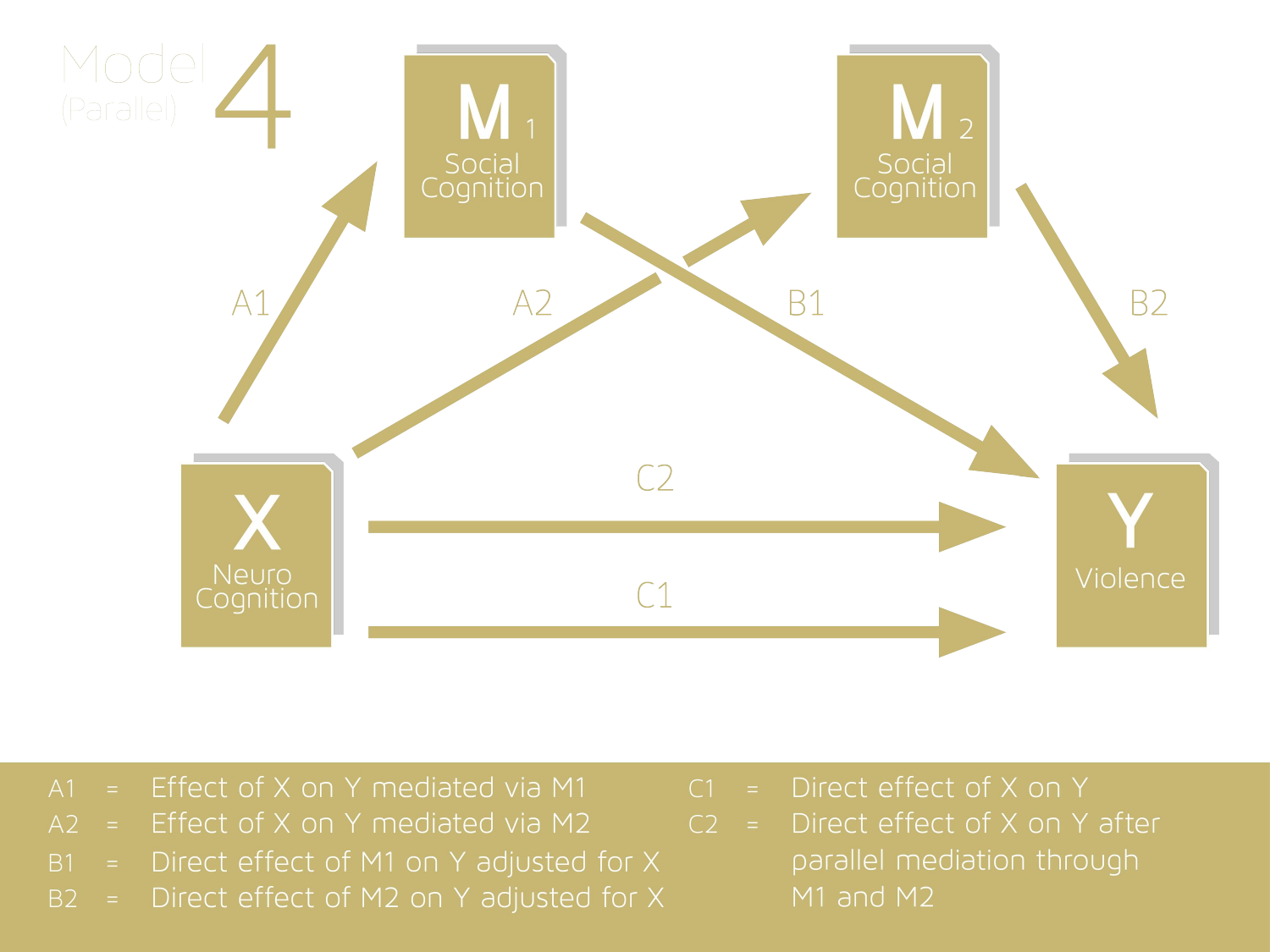

#

## Slide 3
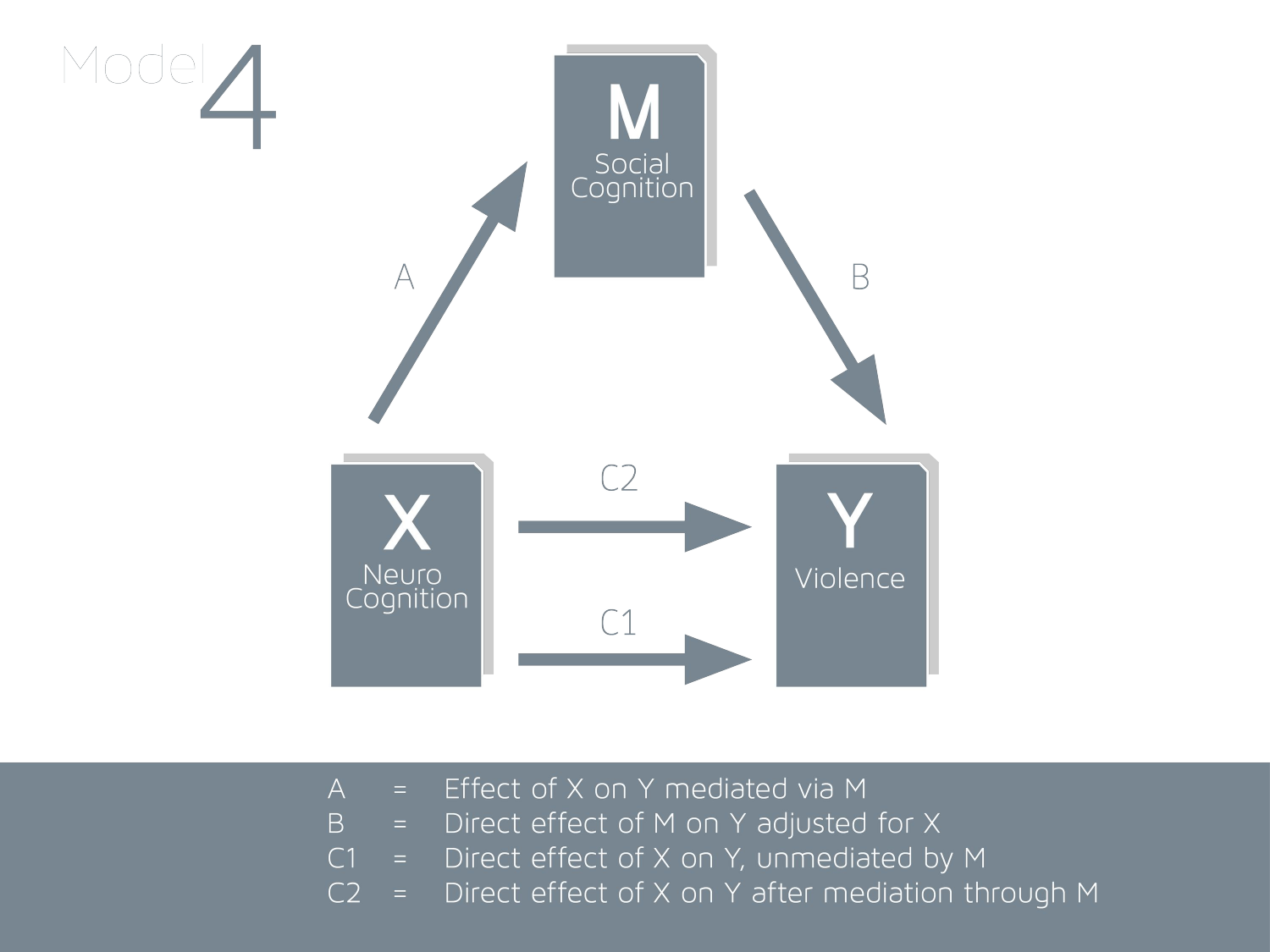

#

## Slide 4
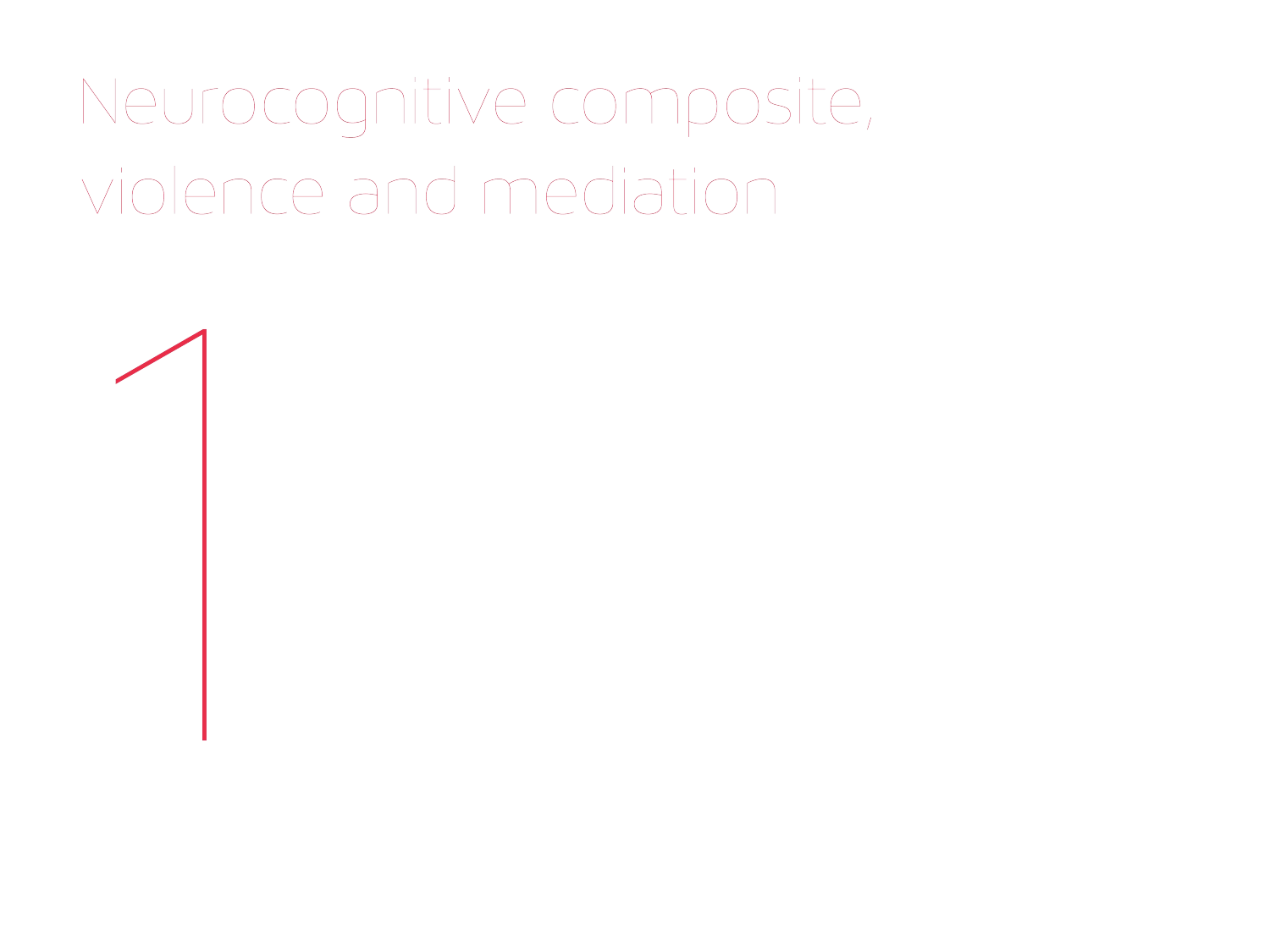

#

## Slide 5
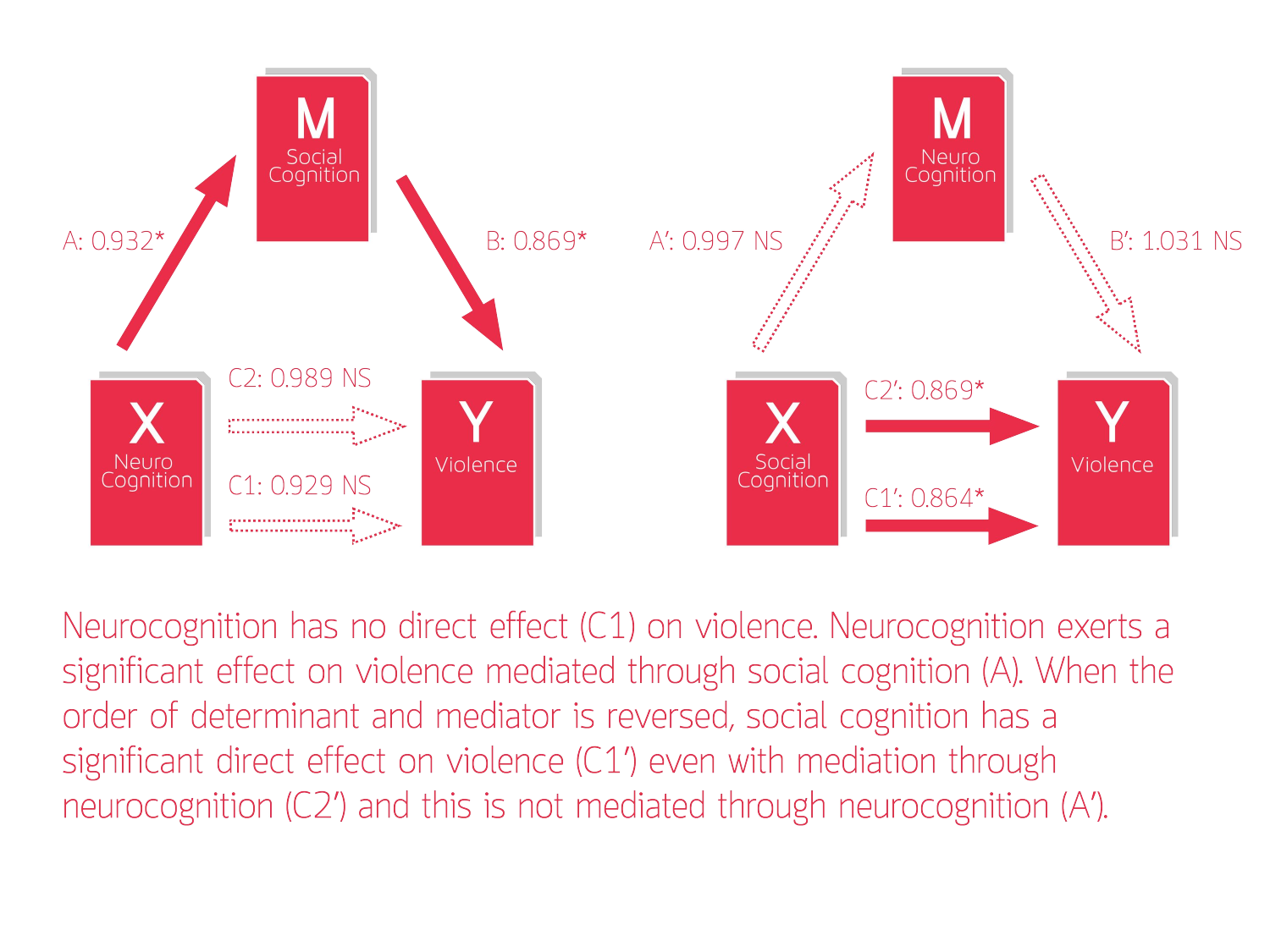

#

## Slide 6
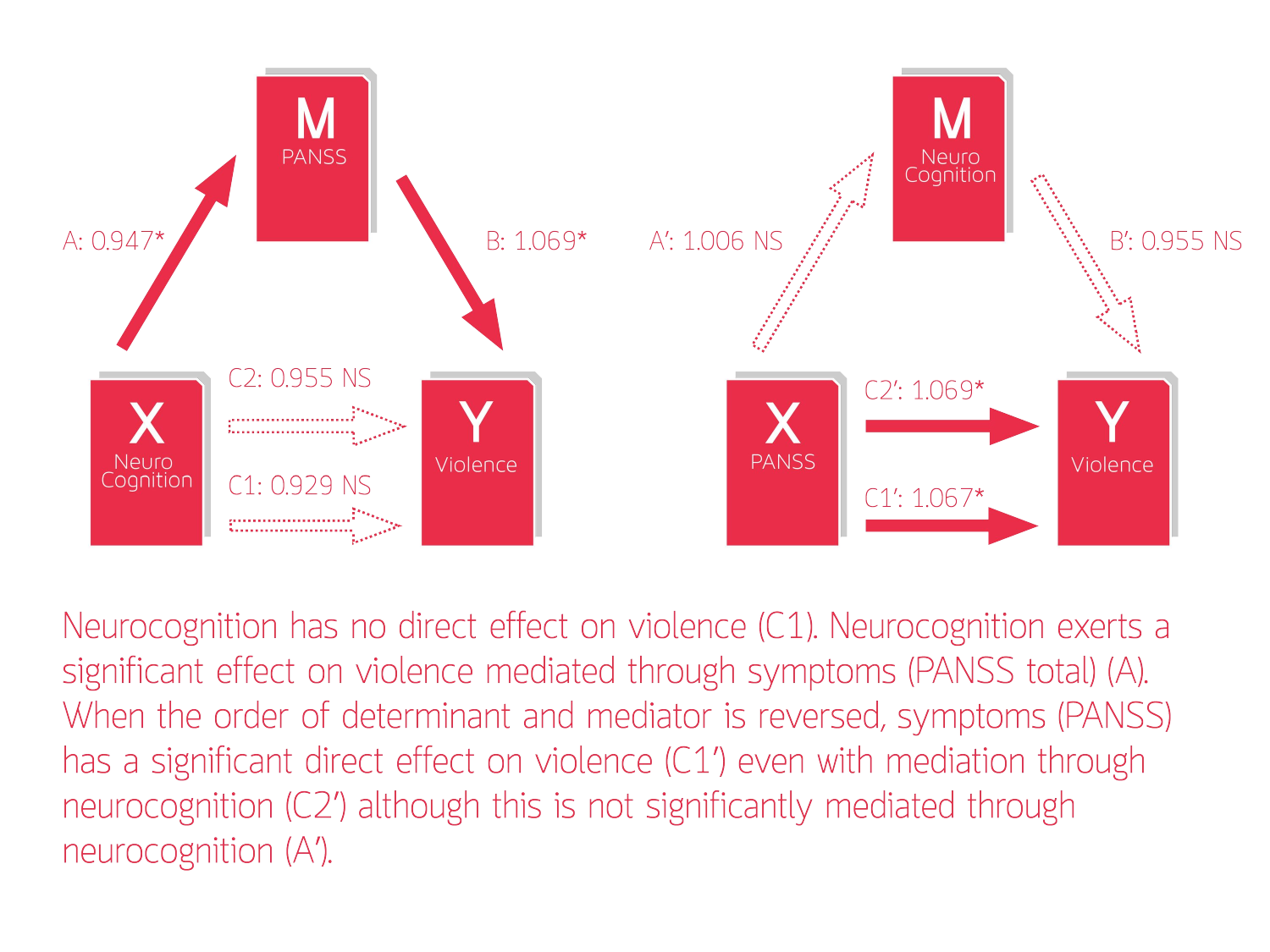

#

## Slide 7
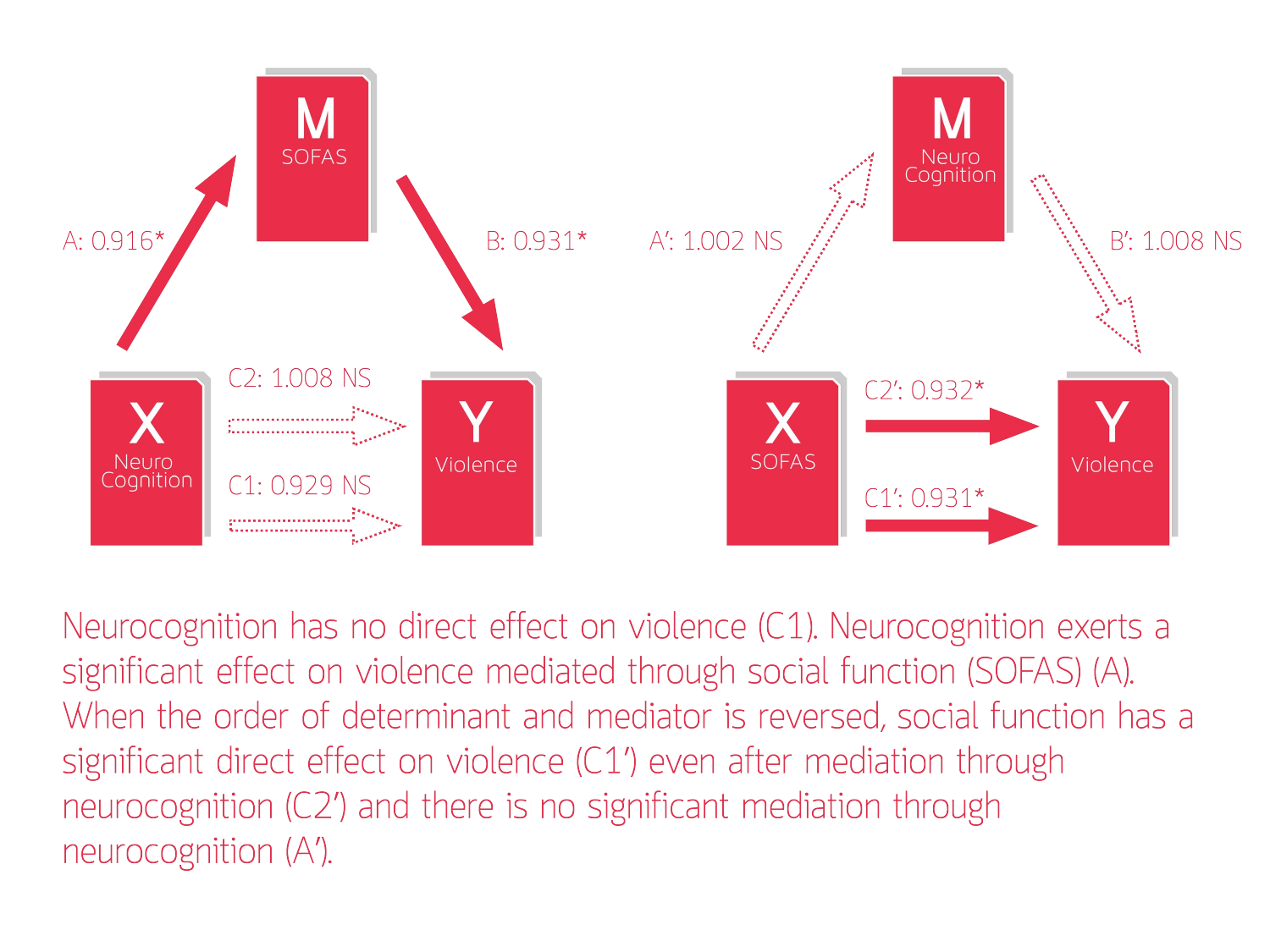

#

## Slide 8
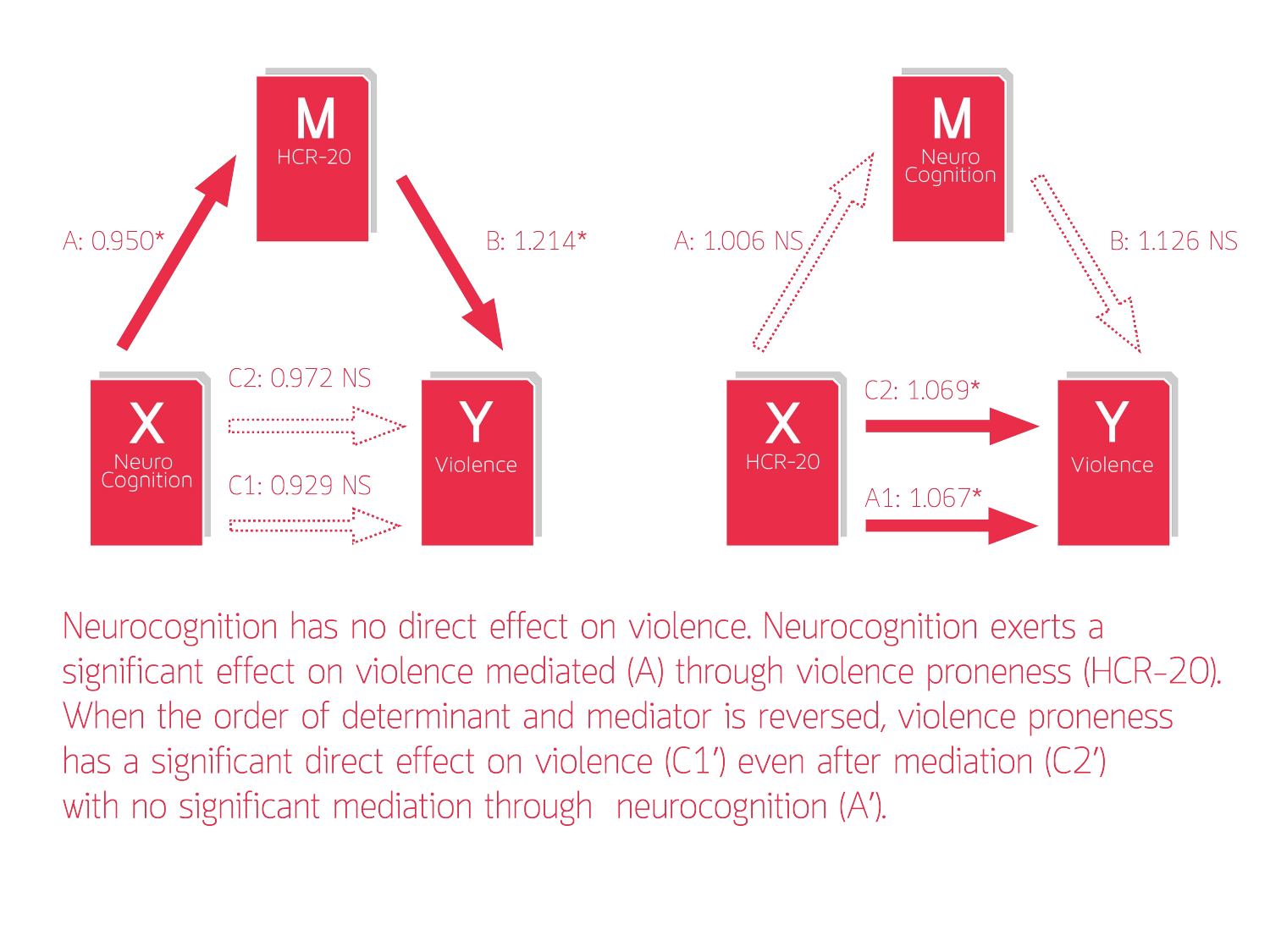

#

## Slide 9
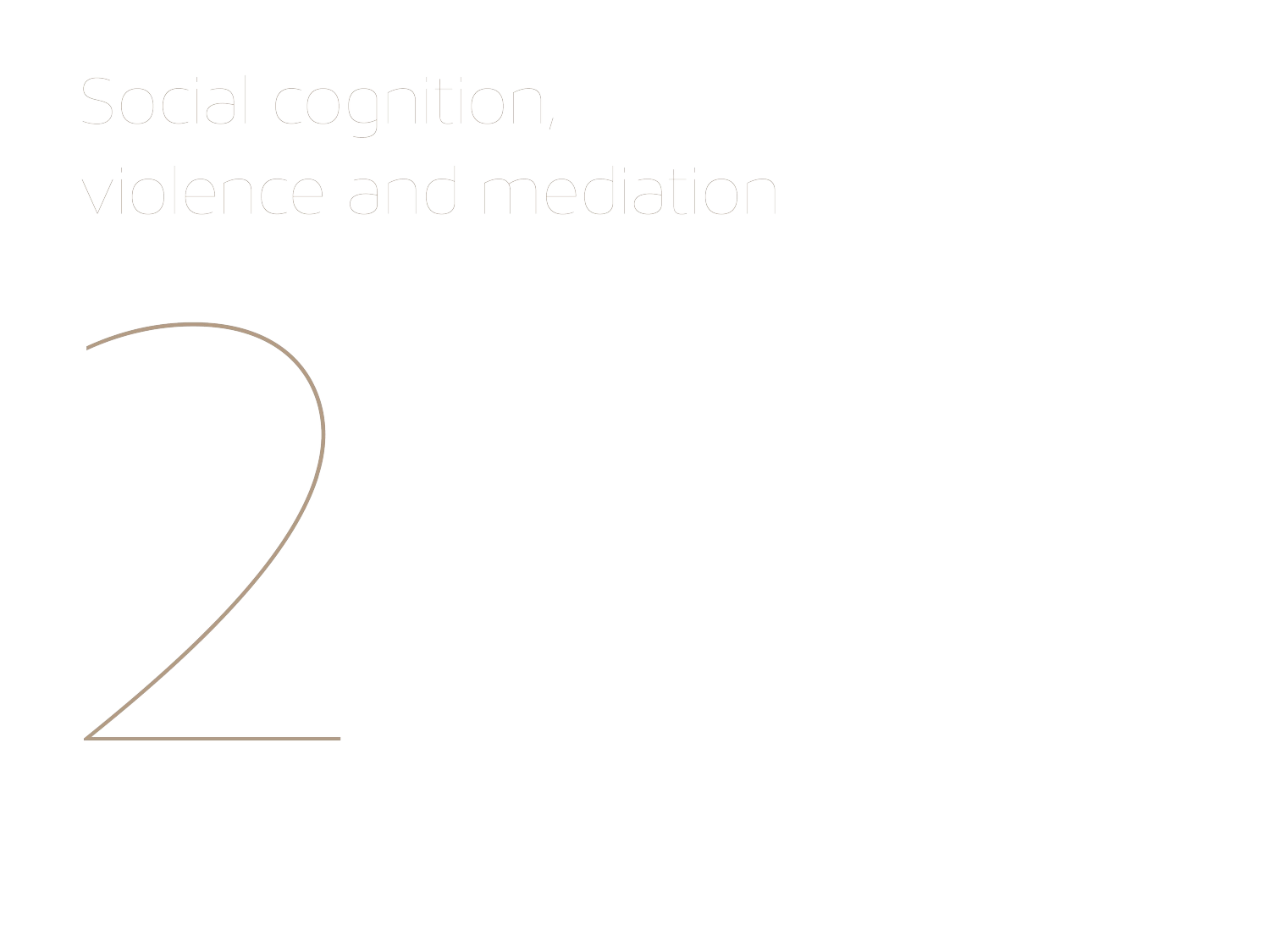

#

## Slide 10
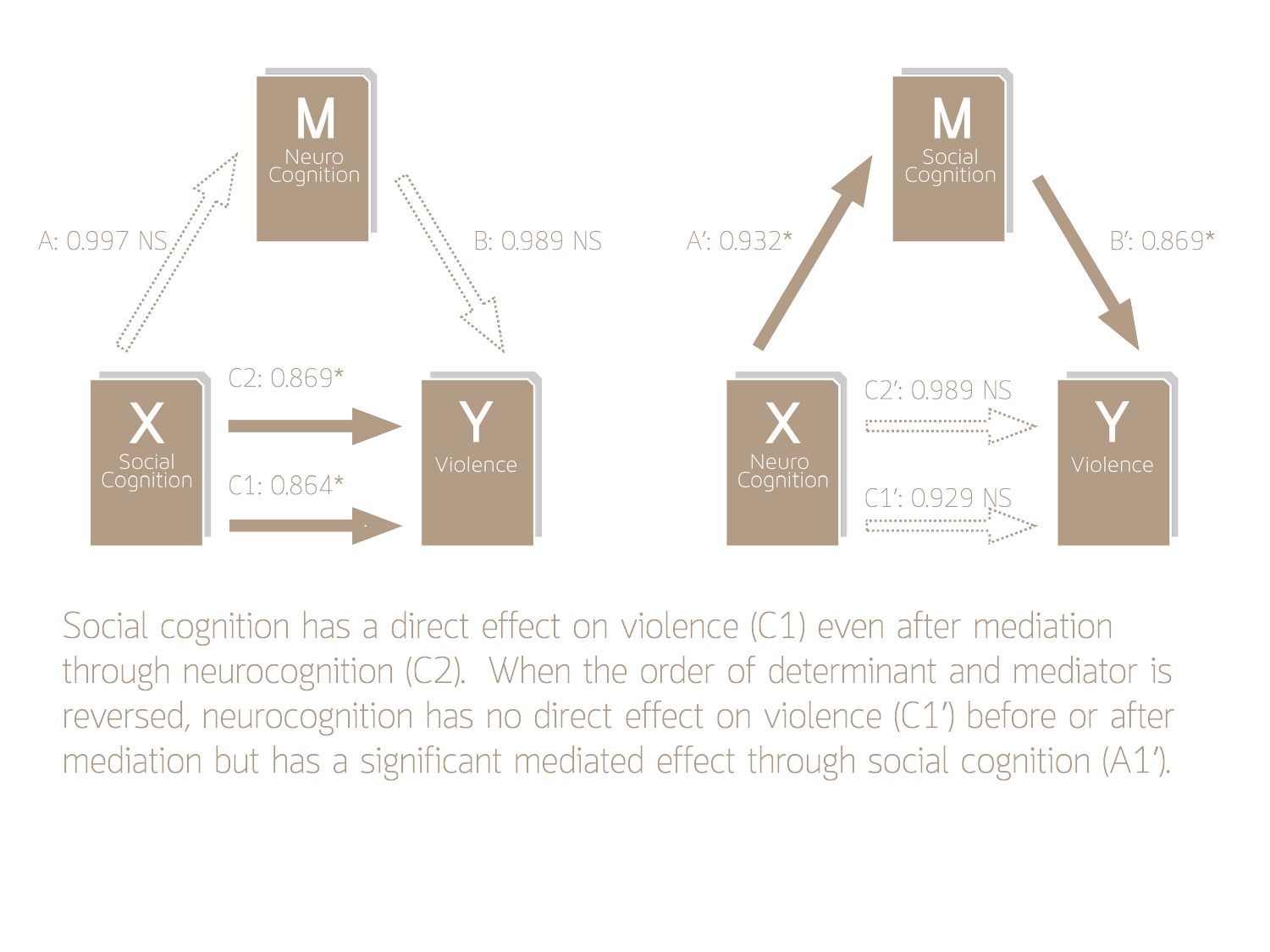

#

## Slide 11
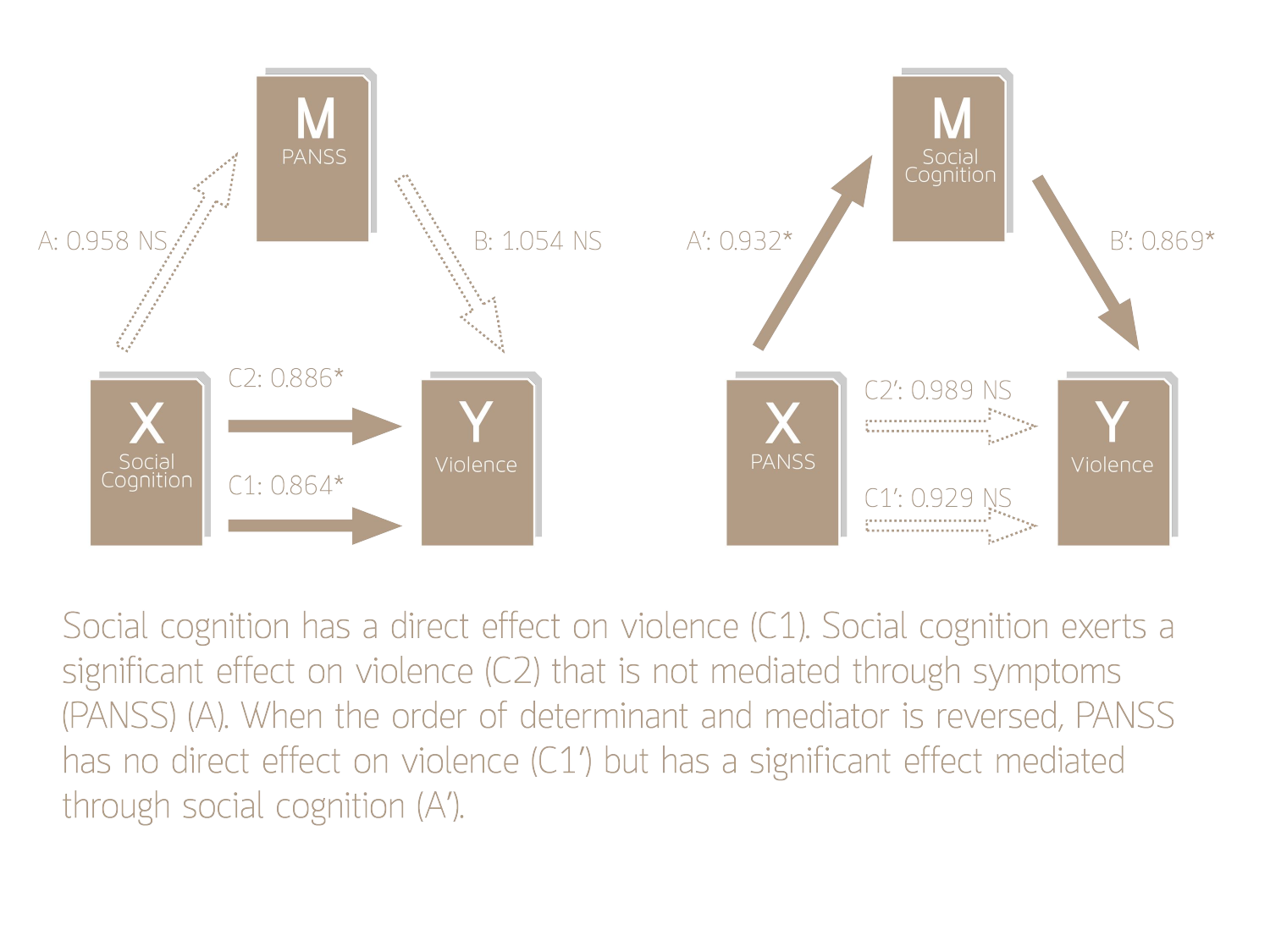

#

## Slide 12
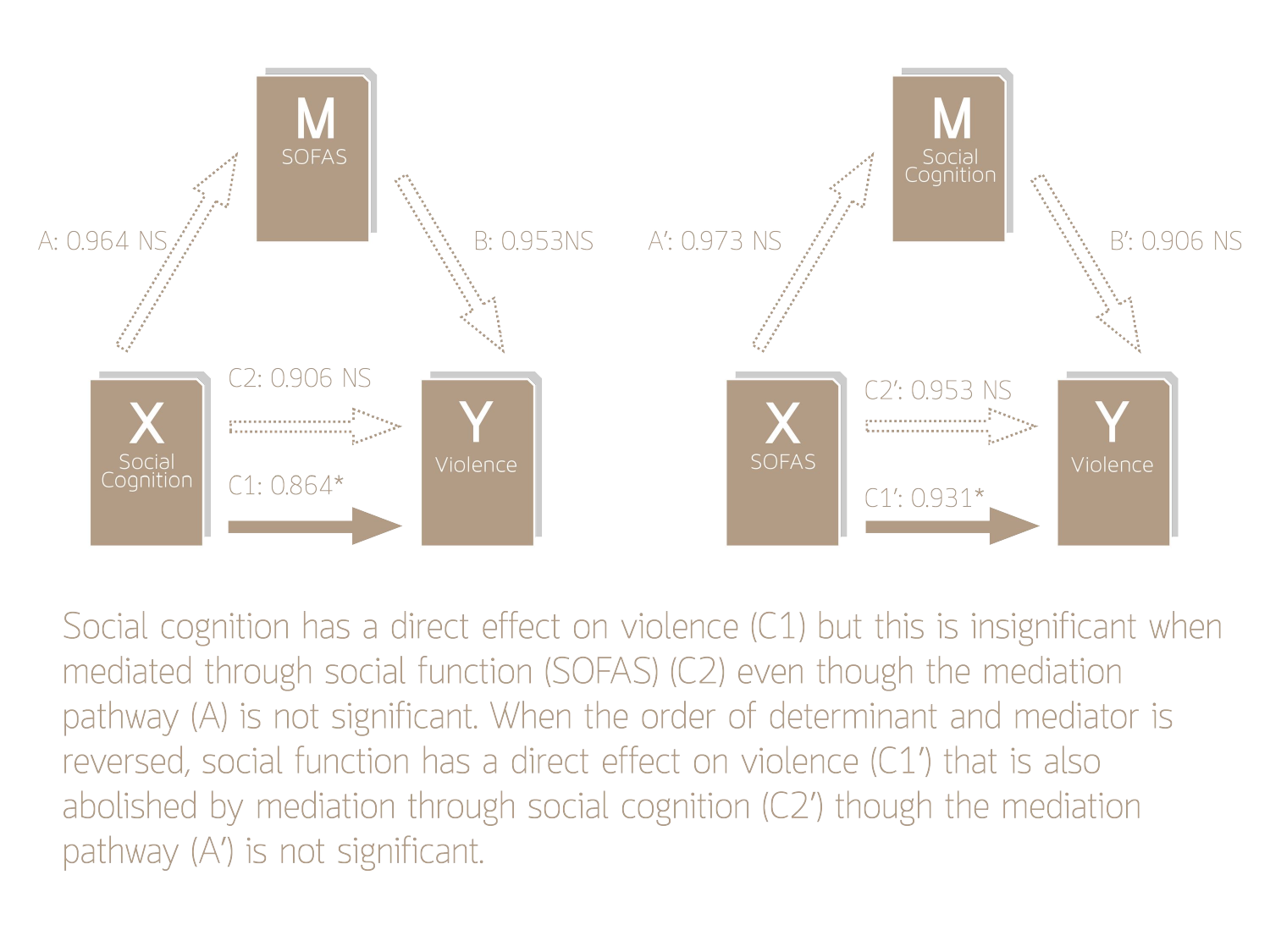

#

## Slide 13
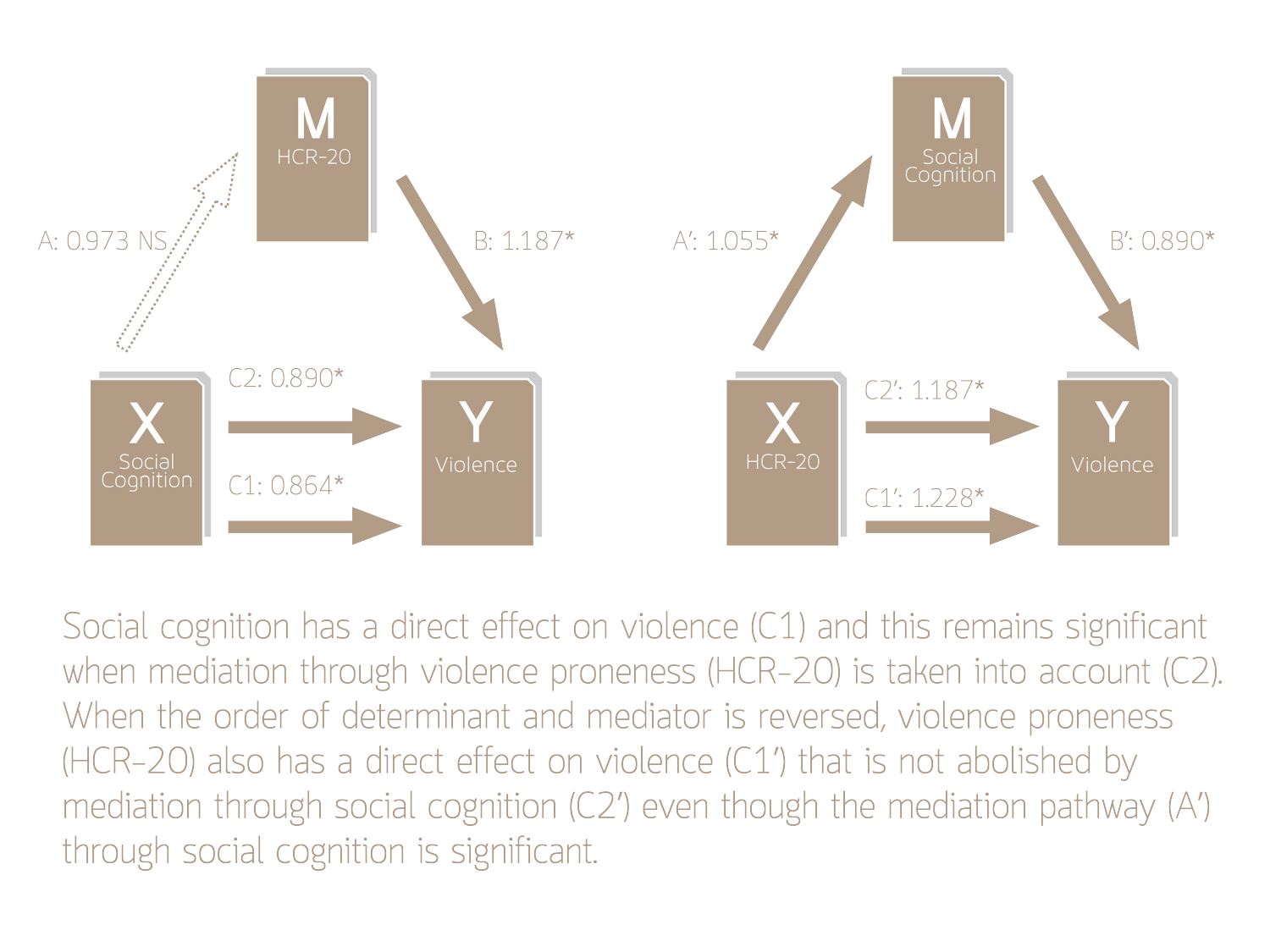

#

## Slide 14
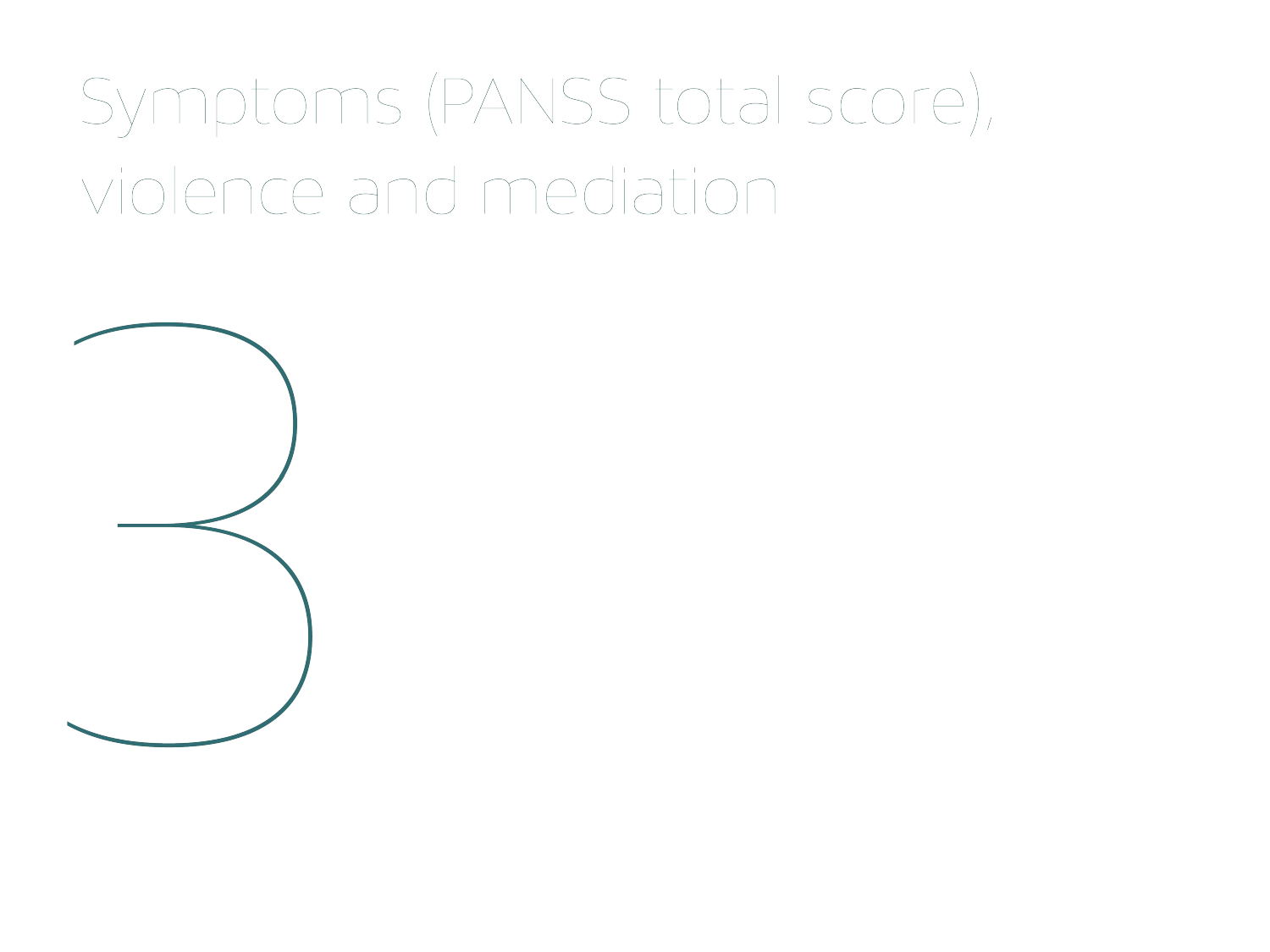

#

## Slide 15
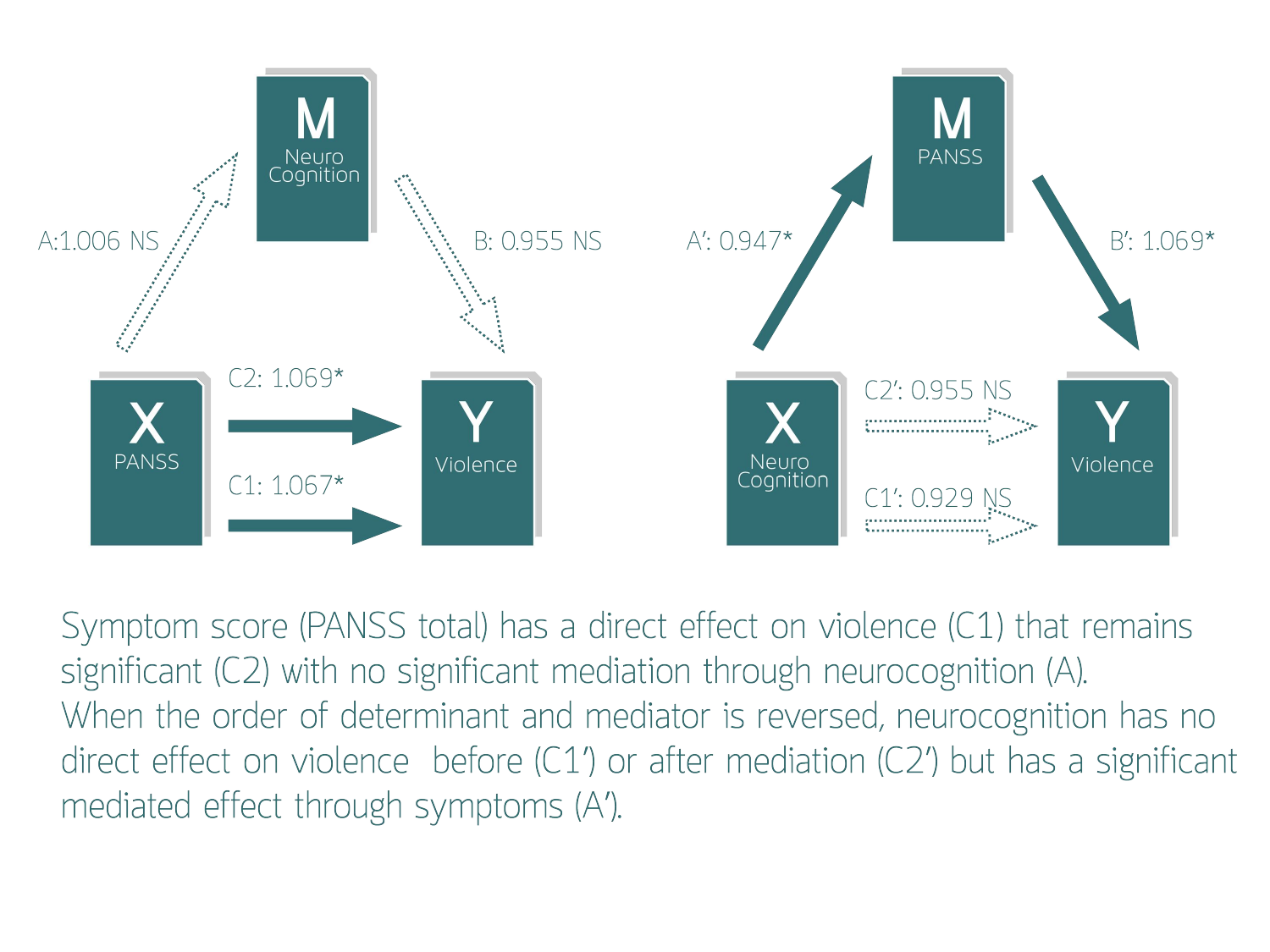

#

## Slide 16
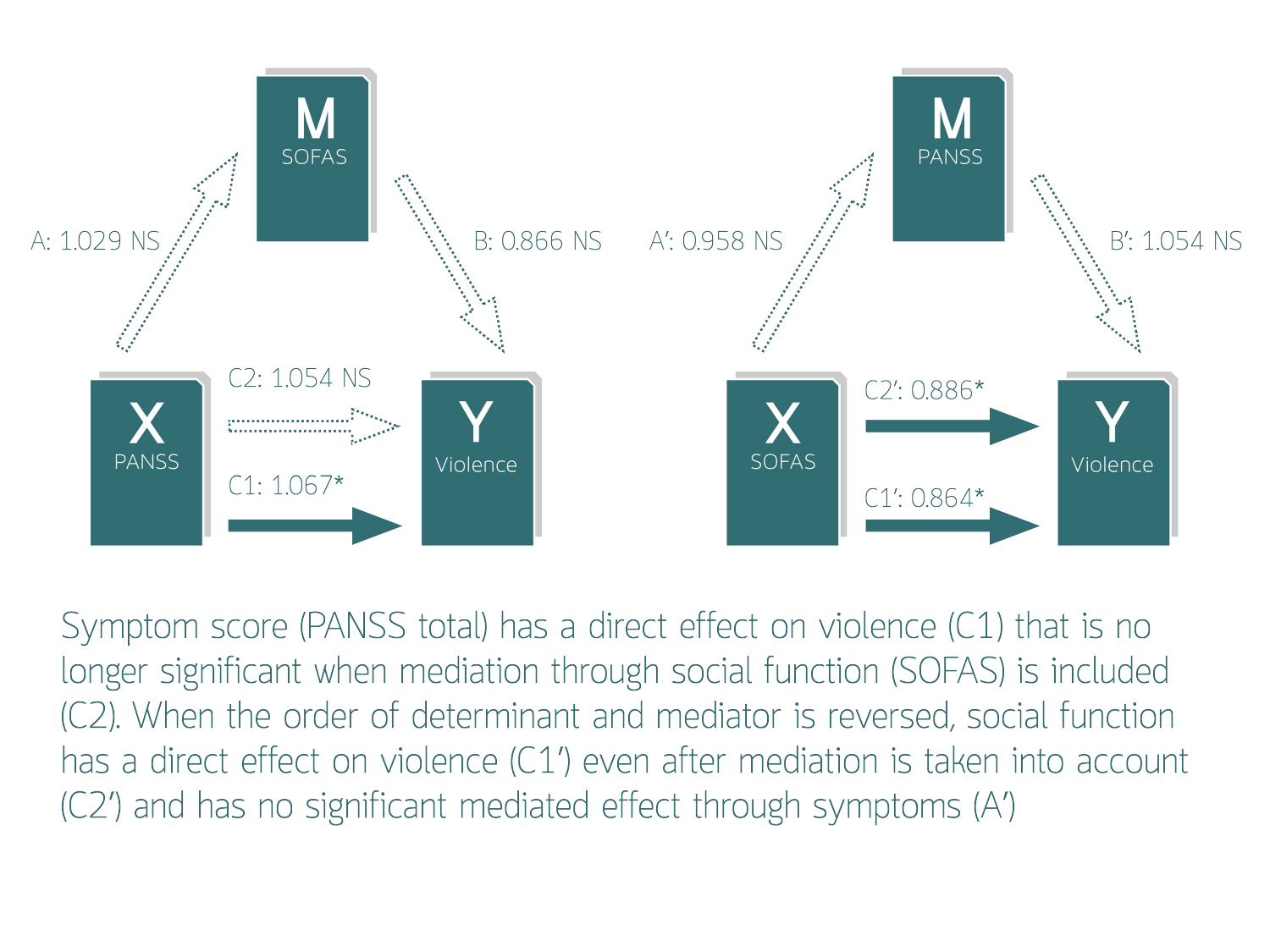

#

## Slide 17
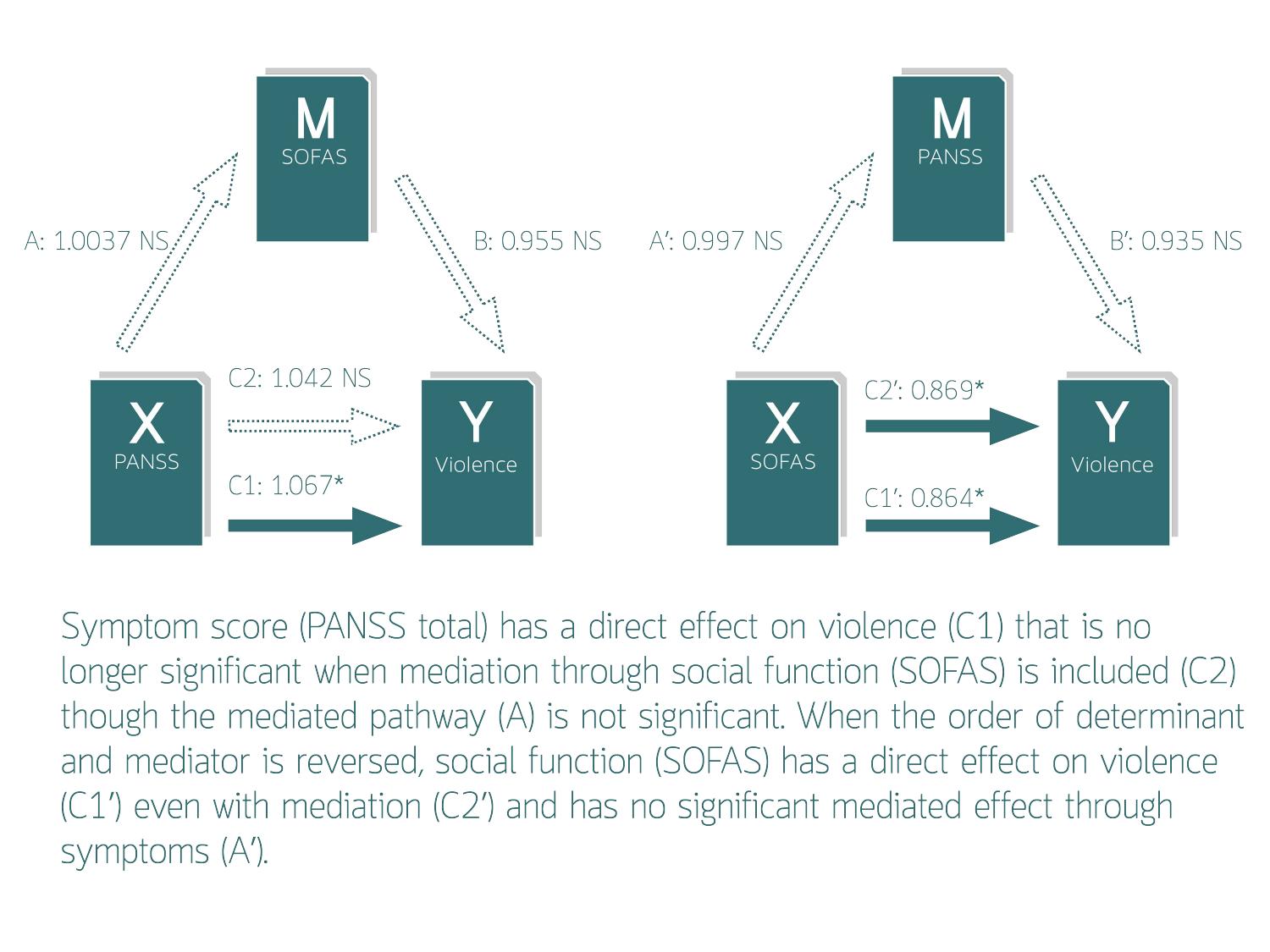

#

## Slide 18
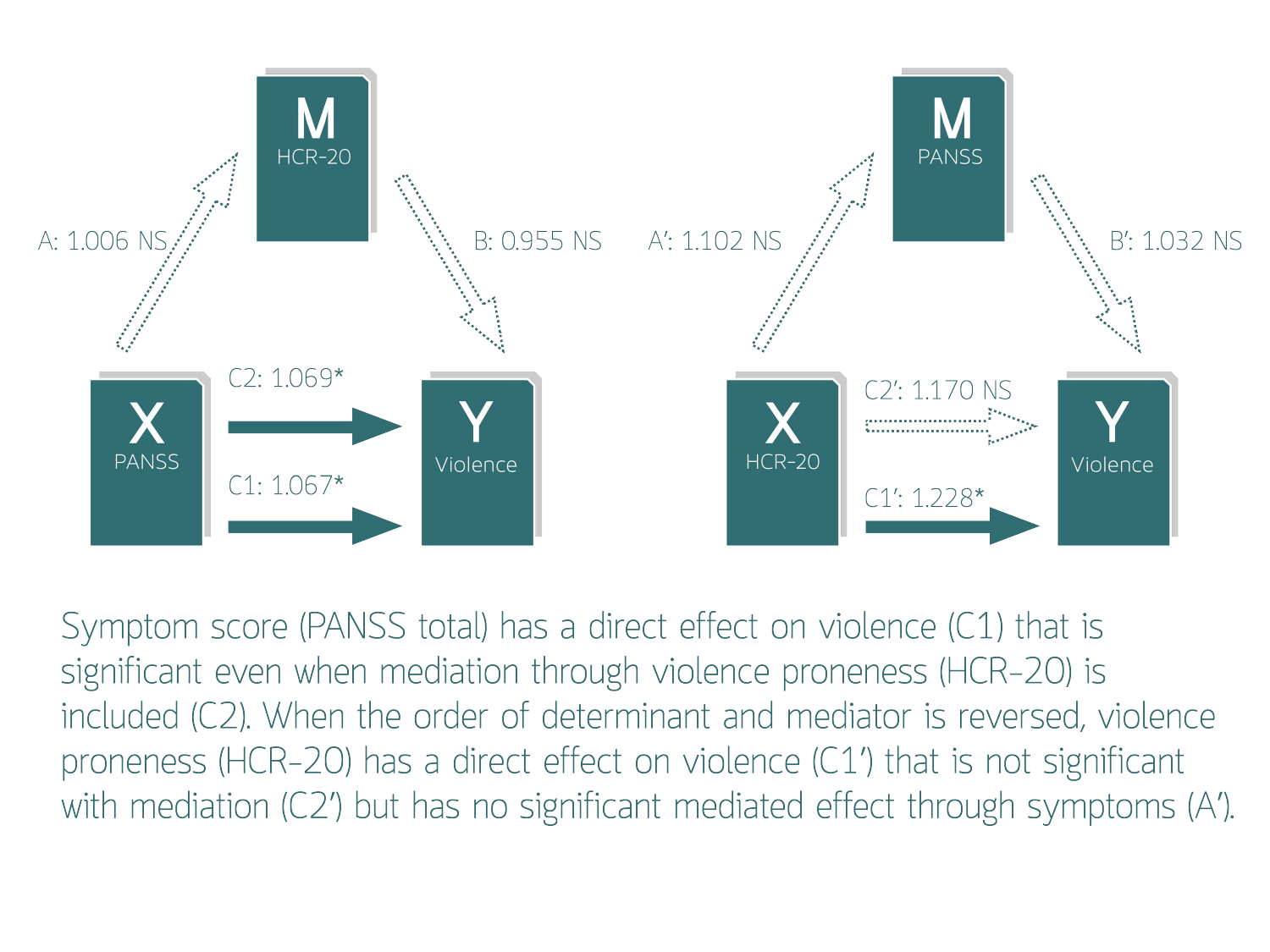

#

## Slide 19
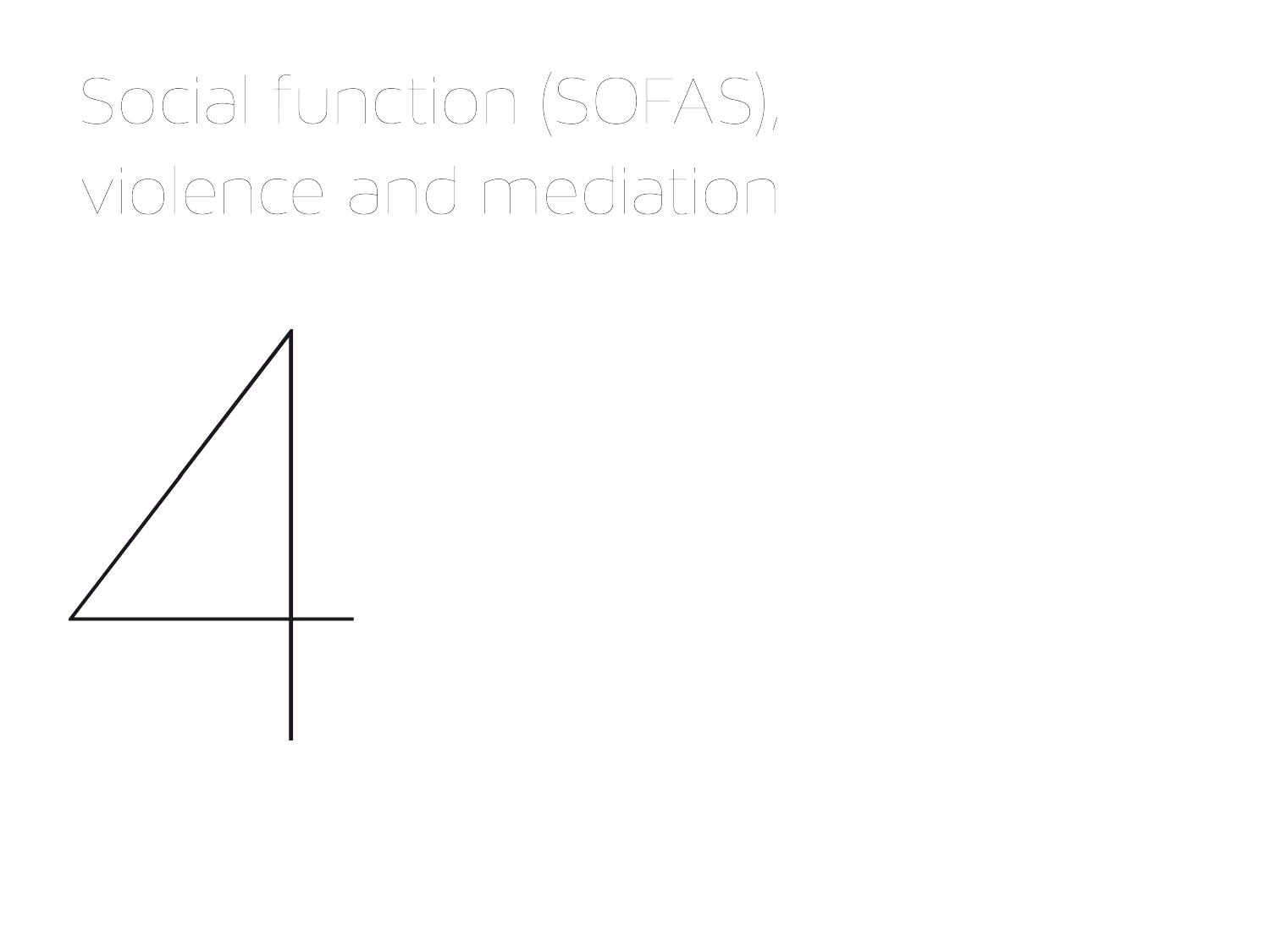

#

## Slide 20
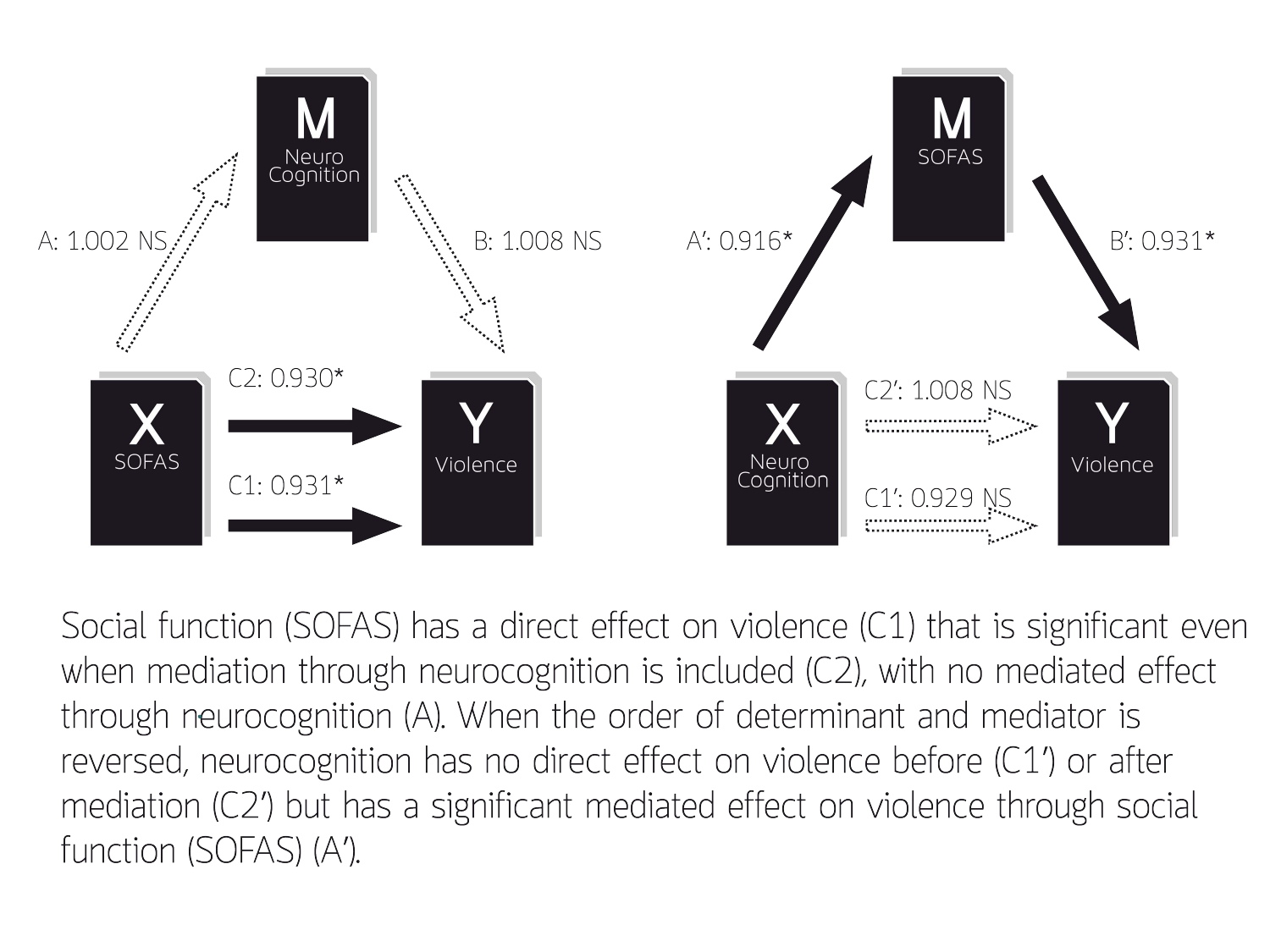

#

## Slide 21
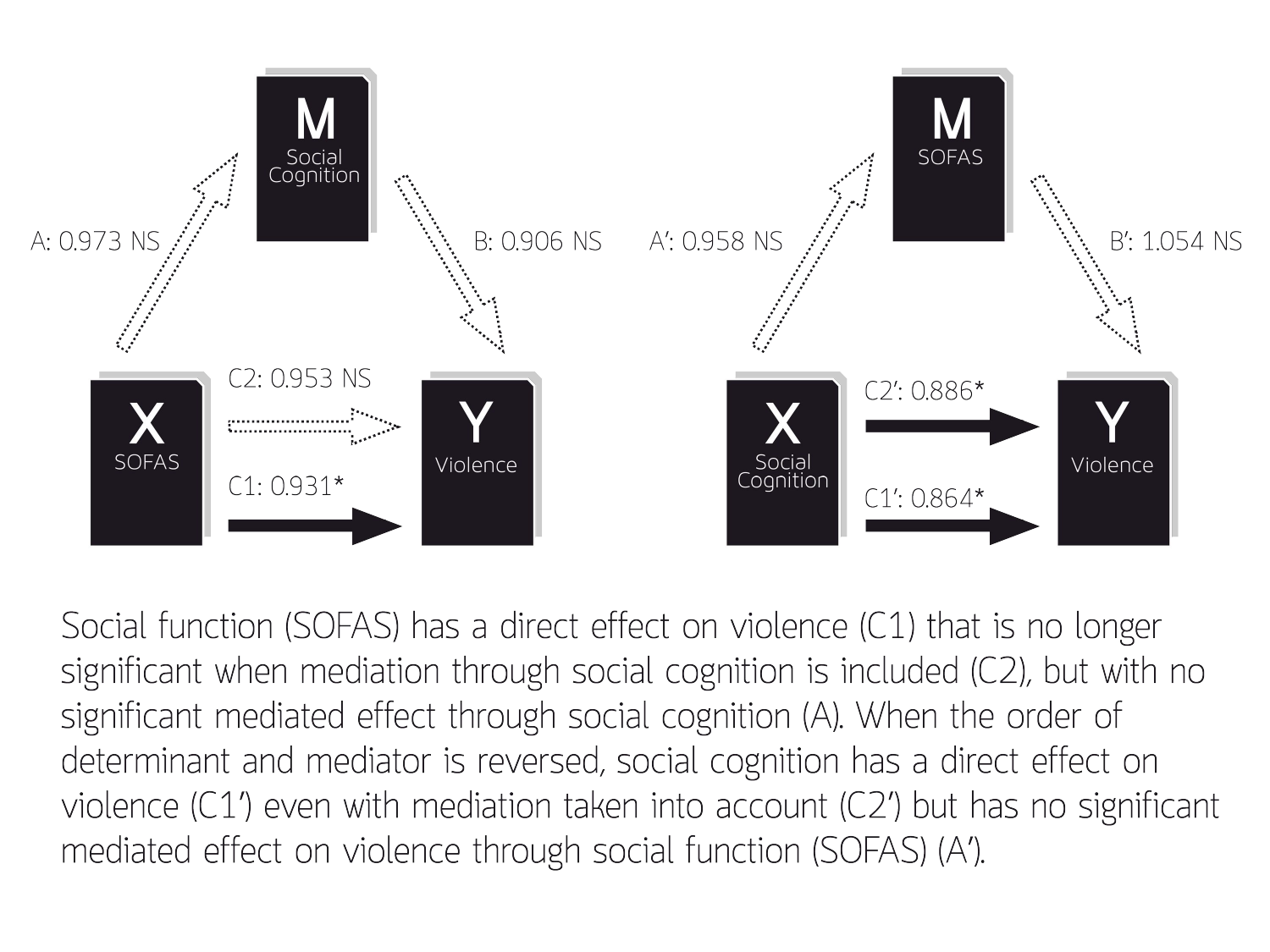

#

## Slide 22
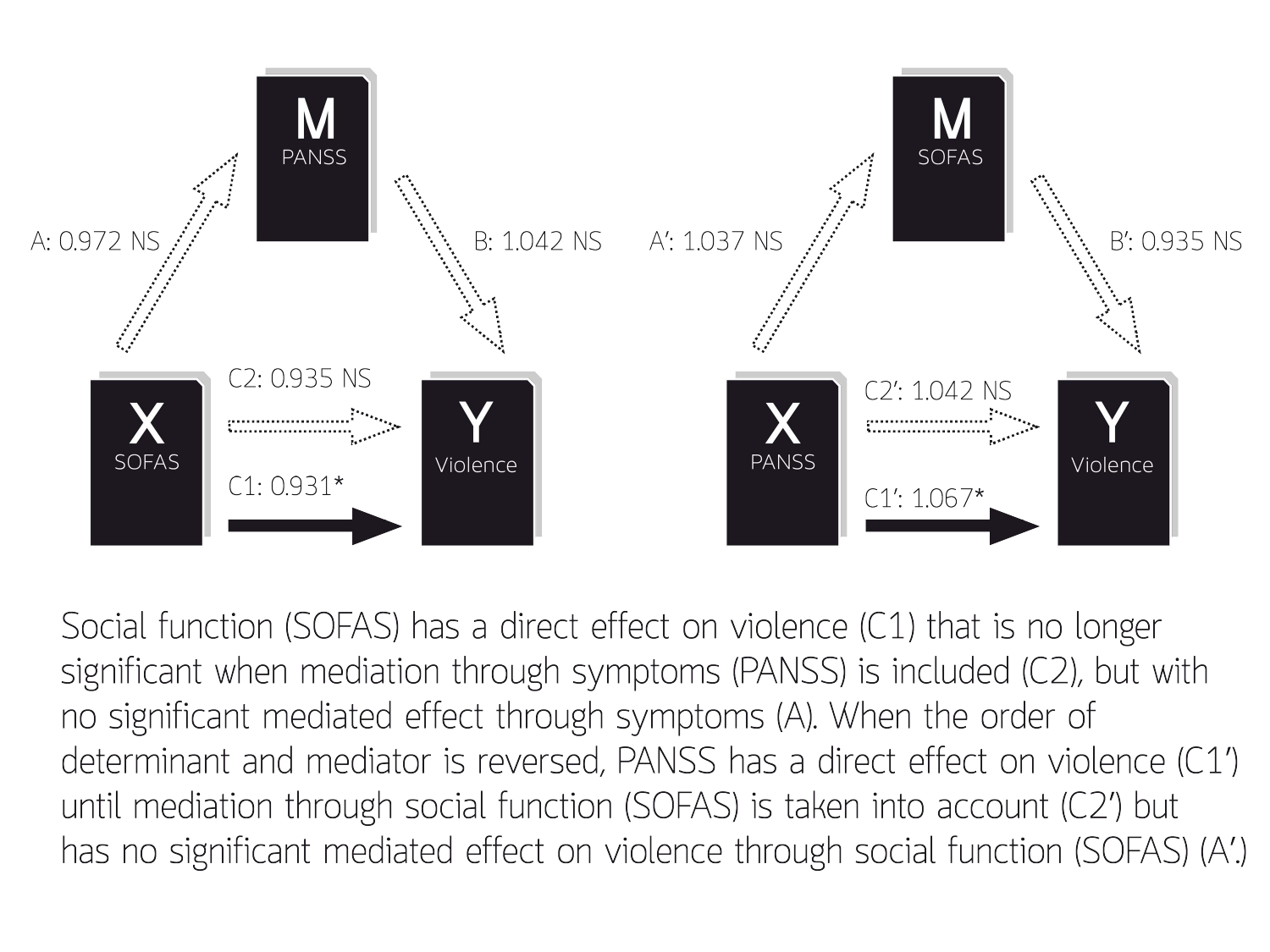

#

## Slide 23
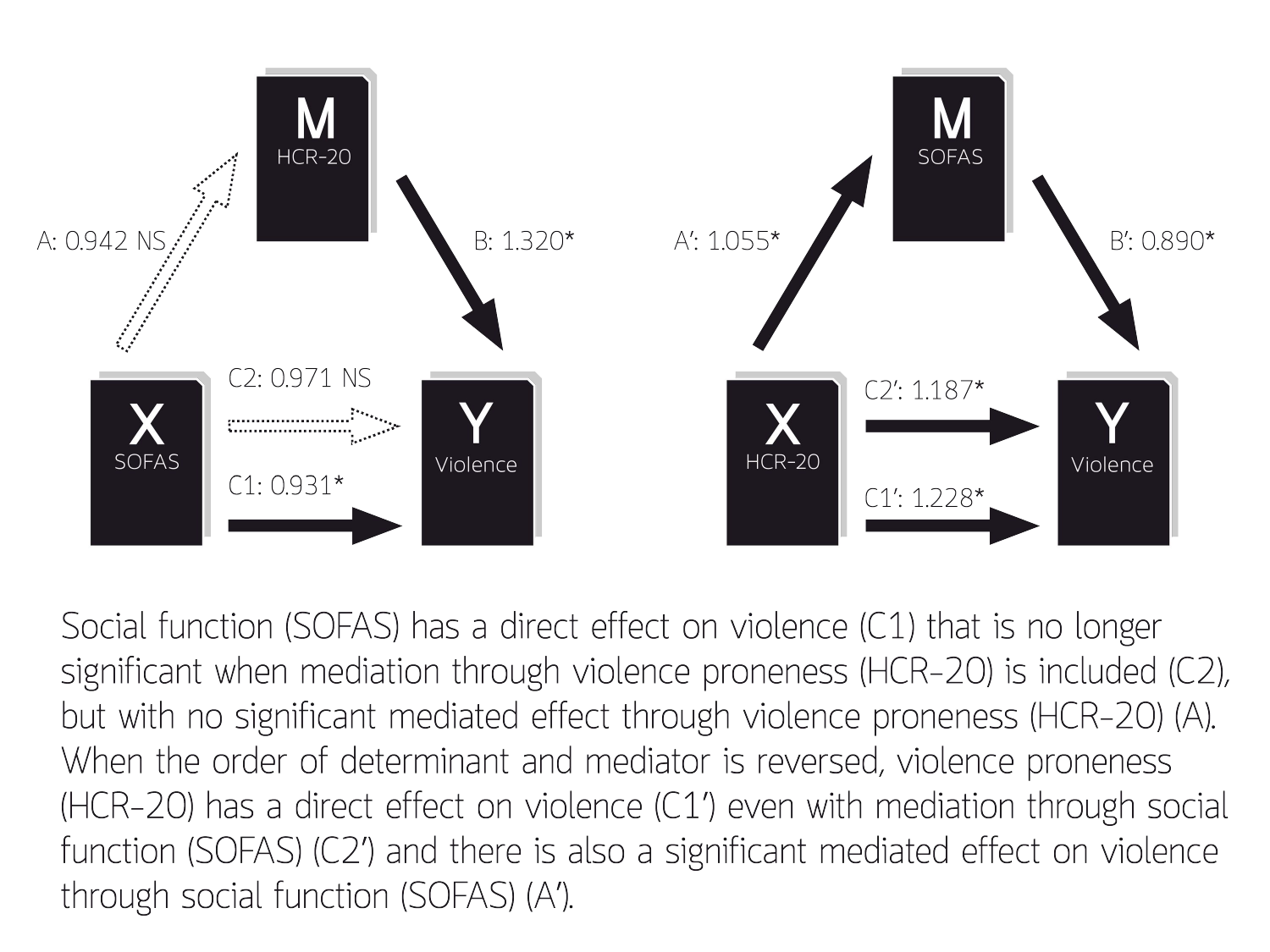

#

## Slide 24
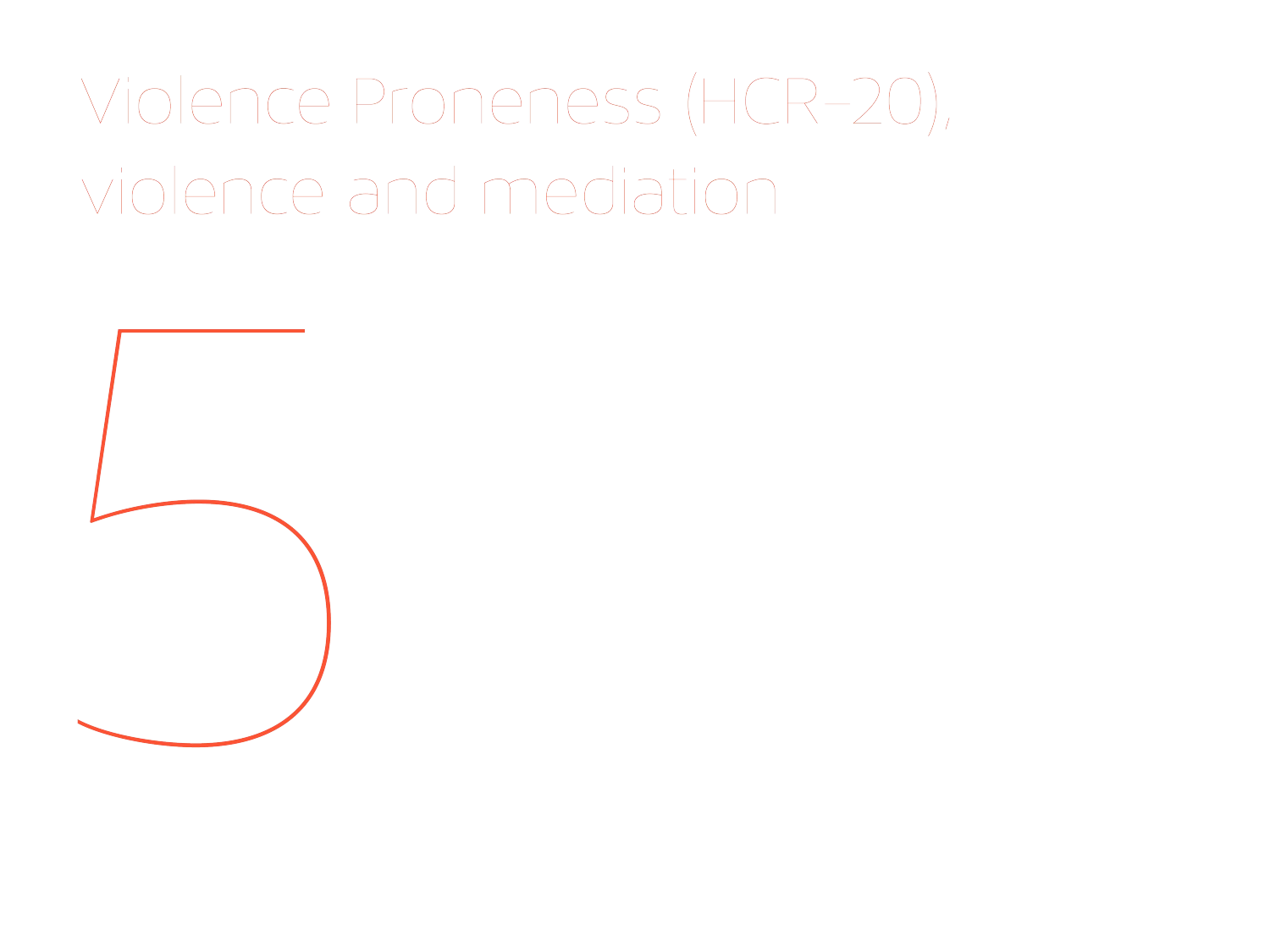

#

## Slide 25
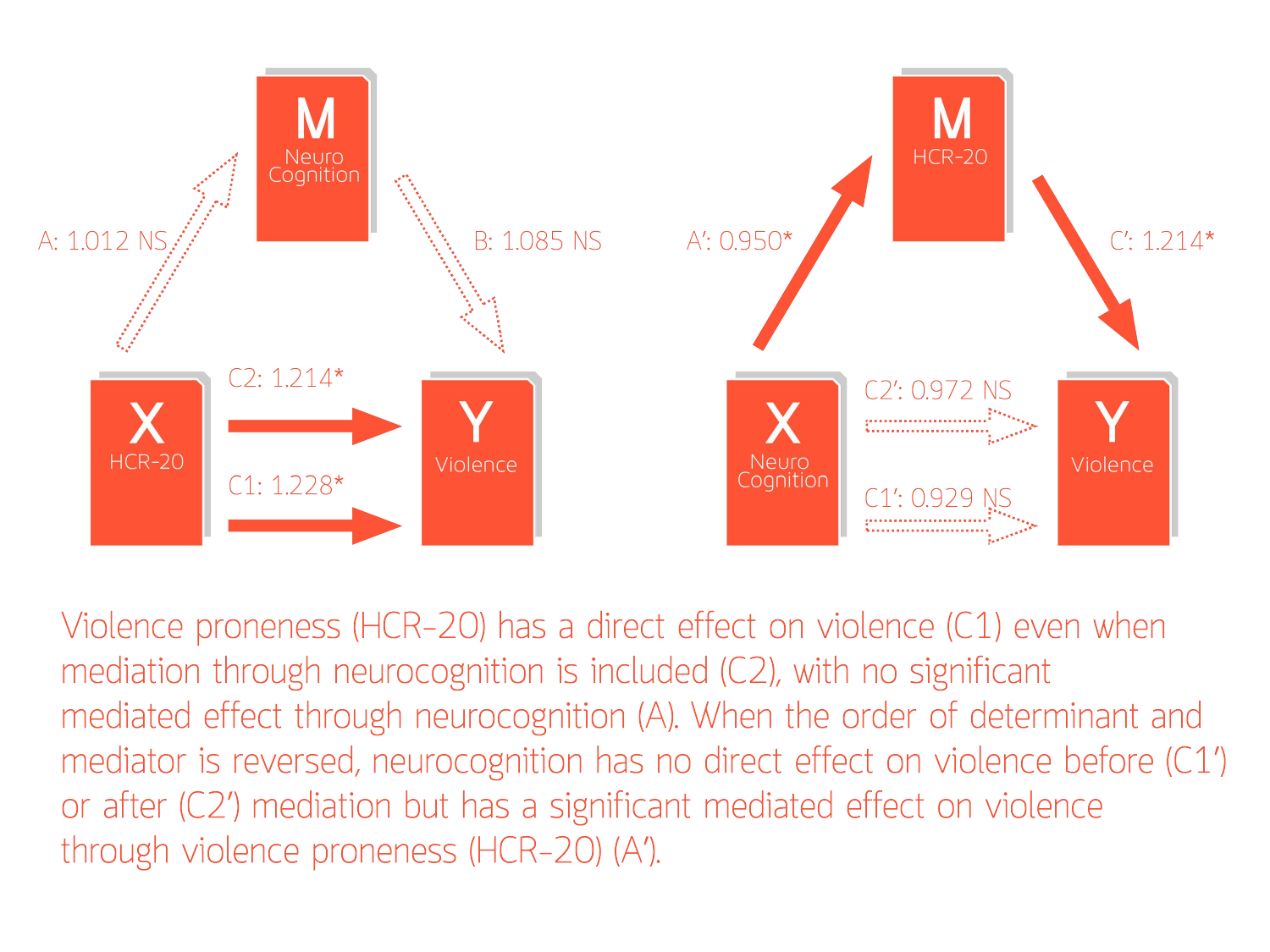

#

## Slide 26
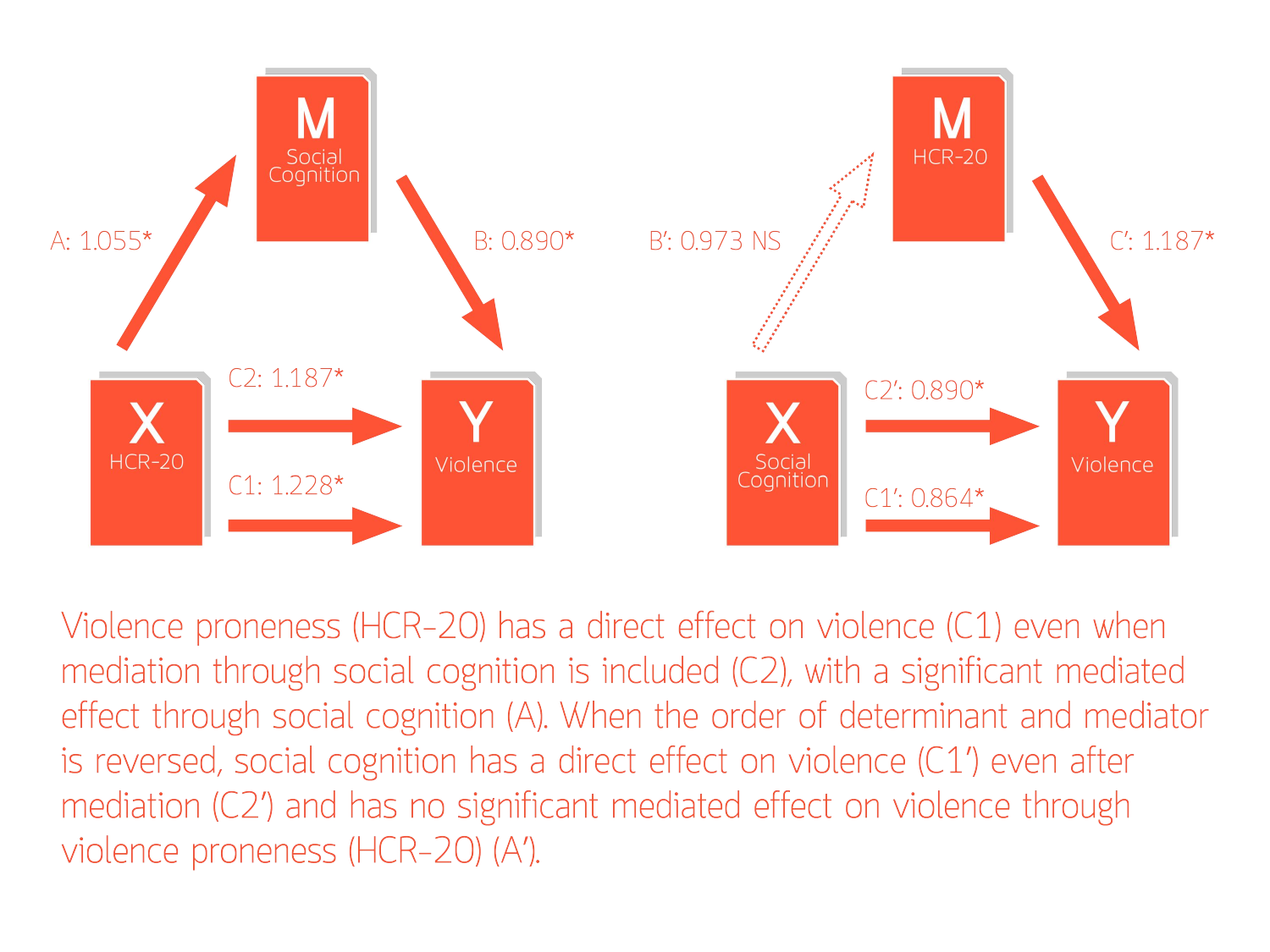

#

## Slide 27
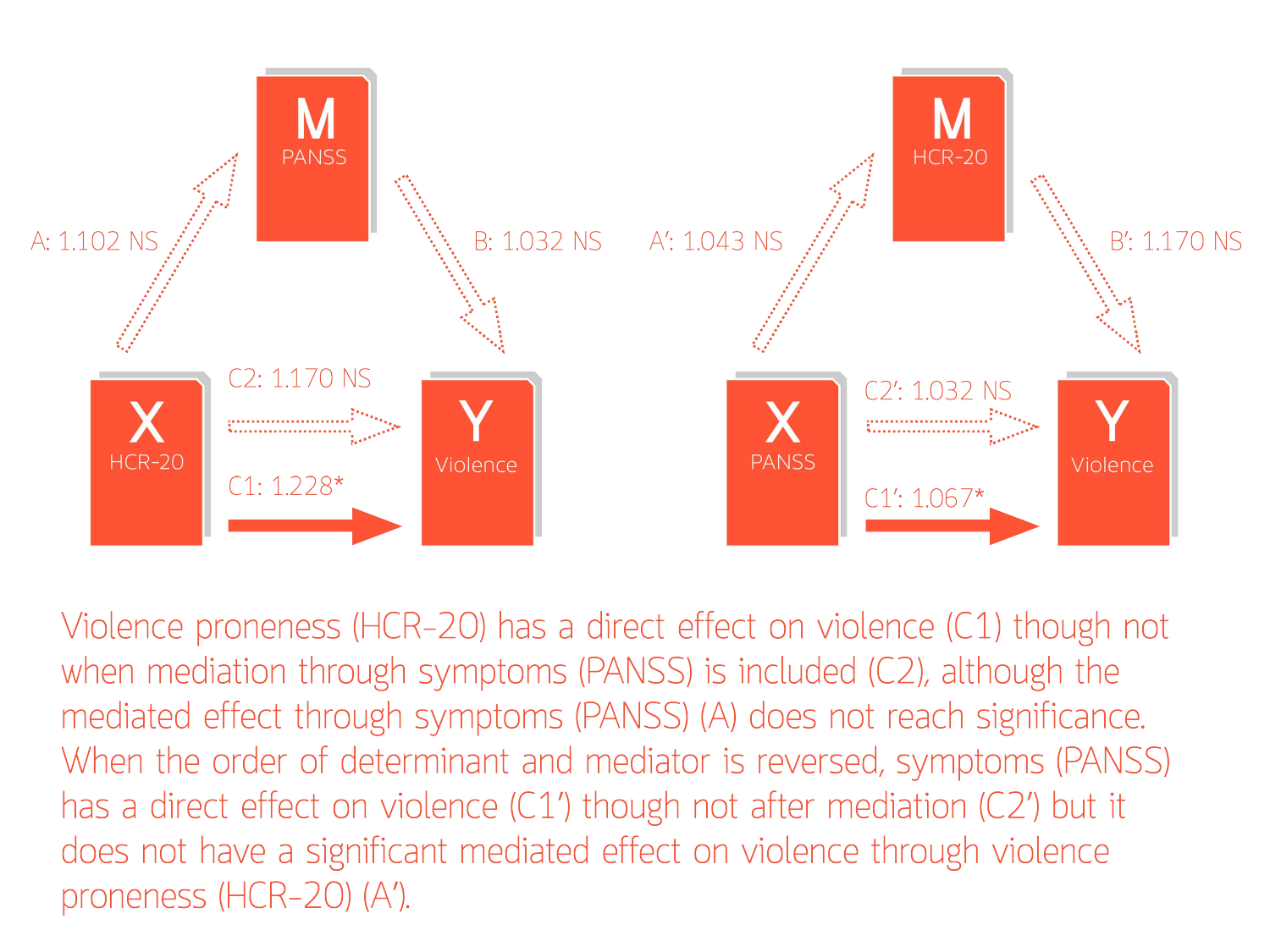

#

## Slide 28
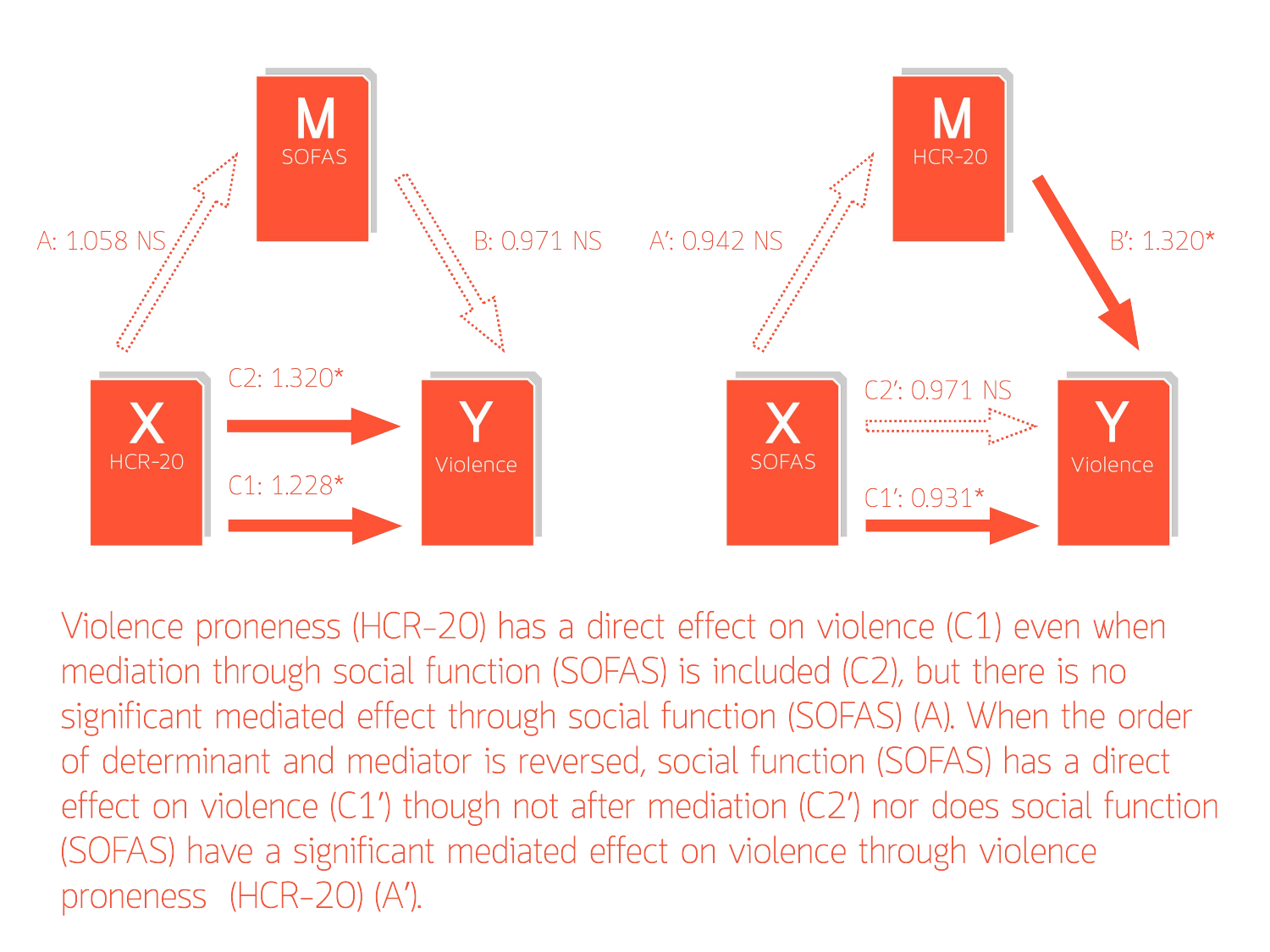

#
